# Completion Rates of Food Frequency Questionnaires and Food Records in People with Chronic Conditions: Systematic Review and Meta-Analysis

**DOI:** 10.3390/nu18121922

**Published:** 2026-06-13

**Authors:** Amanda Kyei, Chiara Miglioretto, Geraldine Perez, Kelly Lambert

**Affiliations:** 1 School of Medical, Indigenous and Health Sciences, University of Wollongong, Wollongong, NSW 2500, Australia; amanda.a.kyei@gmail.com (A.K.); chiaram@uow.edu.au (C.M.); geraldine@thegutfriendlydietitian.com.au (G.P.); 2 Health Innovations, University of Wollongong, Wollongong, NSW 2500, Australia; 3 Kidney Lifestyle Research Group, University of Wollongong, Wollongong, NSW 2500, Australia

**Keywords:** systematic review, meta-analysis, observational study, dietary assessment, food record, food frequency questionnaire, chronic disease

## Abstract

**Background/Objectives**: Dietary assessment tools are essential for quantifying food and nutrient intake, characterising dietary patterns, and informing nutrition research. Food Frequency Questionnaires (FFQs) and Food Records (FRs) are widely implemented in observational studies, but completion rates vary, which may compromise data quality, introduce bias, and limit the interpretation of findings. This review is intended to synthesise evidence from observational studies on completion rates of these tools in populations with chronic conditions. **Methods**: A systematic search of Medline, PubMed, Scopus, and Google Scholar was completed. Eligible studies were observational studies using an FFQ or FR published from January 2015 to May 2025 in people with a chronic condition. Methodological quality was evaluated using the Hoy Risk of Bias tool for observational studies of prevalence. Subgroup meta-analyses estimated pooled mean completion proportions with 95% confidence intervals, and heterogeneity was assessed using the *I*^2^ statistic. This study was registered with the International Prospective Register of Systematic Reviews and reported according to PRISMA guidelines. **Results**: Of the 8921 records screened, 88 studies (*n* = 84,579 participants) met inclusion criteria. The combined FFQ and food record mean pooled completion rate was 79.1% (95% Confidence Interval (CI): 74.37–83.43%). However, substantial heterogeneity was observed, indicating considerable variability across studies. Subgroup analyses highlighted important differences by tool type, format, age group, and disease category. FFQs demonstrated higher completion rates (80.6%) than FRs (74.3%). Electronic formats had higher completion rates than paper formats. Completion rates were higher in adults than in pediatric cohorts, and varied by chronic condition type, with kidney disease associated with the highest completion rates. **Conclusions**: These findings highlight the importance of considering tailored dietary data collection strategies, particularly for paediatric and medically complex populations, and provide direction for enhancing the feasibility of dietary assessment collection in future research.

## 1. Introduction

Dietary assessment is central to nutrition research and clinical practice, and underpins the accurate evaluation of dietary intake, identification of nutritional risk, and development of targeted interventions across the life course. In people living with chronic conditions such as diabetes, kidney disease, cancer and cardiometabolic conditions, accurate dietary data are critical due to associations between diet and disease progression, symptom burden, and long-term health outcomes [1,2]. Globally, the prevalence of chronic disease is increasing [3], with multimorbidity now common in many adult populations [4]. In Australia, nearly half of adults live with at least one chronic condition [5], and a substantial proportion of children and adolescents are also impacted, often with long-term health implications extending into adulthood [6].

Food frequency questionnaires (FFQs) and food records (FRs) are among the most widely used self-reported dietary assessment tools in observational research [7]. FFQs are designed to estimate habitual intake over extended periods and are valued for their efficiency and scalability in epidemiological studies but at the cost of increased participant burden. In contrast, FRs capture detailed short-term intake and are often considered advantageous for reducing recall bias, but at the cost of increased participant burden [8]. These two tools represent contrasting approaches in terms of respondent burden and cognitive demand, making them particularly relevant for evaluating feasibility and data completeness. Despite their widespread application, both tools are vulnerable to incomplete data, and in research and practice, there are variable completion rates across study designs, populations, and modes of delivery [9].

Incomplete dietary data are an important limitation, as this can threaten internal validity, reduce statistical power, and may introduce systematic bias if non-completion is associated with participant characteristics such as age, disease severity, cognitive burden, or socioeconomic disadvantage. Emerging evidence suggests that completion rates differ between tool types and formats [10,11]. For example, recent studies in gastrointestinal [12,13] and metabolic disease populations report markedly lower completion for multi-day food records compared with FFQs, particularly among younger participants [14] and those experiencing greater symptom burden [15]. Advances in electronic dietary assessment platforms, including mobile applications and web-based surveys, have the potential to mitigate some barriers to completion rates through automated prompts, improved usability, and reduced administrative burden [16]. However, uptake and feasibility remain inconsistent across clinical populations and vary according to digital literacy, access inequality and platform variability [9,17].

These challenges may be amplified in people living with chronic conditions, where competing health priorities, fatigue, treatment demands, and symptom fluctuation are common. Paediatric populations present additional complexity, often requiring proxy reporting by caregivers and navigating family complexity to ensure completion.

While individual studies have reported completion rates for specific tools or populations, no systematic quantitative synthesis of completion rates across FFQs and FRs in chronic disease populations exists. The absence of a comprehensive synthesis limits the ability of clinicians and researchers to make informed decisions about dietary assessment selection and study feasibility in chronic disease contexts. Given this fact, the aim of this systematic review and meta-analysis was to synthesise completion rates of FFQs and FRs used in observational studies involving adults and children living with chronic conditions. Secondary objectives were to examine differences by tool type, format (electronic versus paper-based), age group, disease category, and timing of assessment. By quantifying completion across diverse clinical populations, this review seeks to inform pragmatic, person-centred approaches to dietary data collection in both research and clinical practice.

## 2. Materials and Methods

The review addressed the following question: “What are the completion rates of food records (FRs) or food frequency questionnaires (FFQs) in observational studies involving adults or children living with a chronic condition?” The review protocol was developed from March–April 2025, prior to commencement of the search (May 2025) and was subsequently registered with PROSPERO on 7 October 2025 (CRD420250593124). Completion rates for this study were defined as the proportion of participants who provided usable dietary data according to predefined study criteria (such as fully completed questionnaires or food records meeting minimum reporting requirements).

A comprehensive search strategy was developed in consultation with a medical research librarian. Four databases (Medline, PubMed, Scopus, and Google Scholar) were searched. The search strategy was adapted for each database with search syntax, truncation and Boolean operators modified as required for each platform. Simplified search terms are shown in Table 1 and the full search included Medical Subject Headings (MeSHs) and keywords relating to completion rates, observational study designs, FFQs, FRs, and food diaries. Searches were conducted using combinations of controlled vocabulary (e.g., MeSH terms) and free-text keywords related to dietary assessment tools (e.g., “food frequency questionnaire”, “food record”, “diet diary”), completion (e.g., “completion rate”, “response”, “adherence”), and observational study designs. Boolean operators (AND/OR) and truncation were applied to combine concepts and optimise sensitivity. Search syntax was adapted for each database. The MEDLINE search strategy is provided in Supplementary Table S1. Grey literature was not systematically searched; however, Google Scholar was included as a supplementary source to enhance the sensitivity of the search and capture additional potentially relevant studies. The Harzing ‘Publish or Perish’ interface was used to enable a structured retrieval and export of results from Google Scholar. Results were sorted by relevance. The first 1000 records were screened. Screening was ceased after this predefined limit, as additional results were considered unlikely to yield further eligible studies.

All databases were searched for eligible studies published between 1 January 2015 and May 2025. The search was restricted to studies published from January 2015 onwards to reflect contemporary dietary assessment practices, including increasing use of electronic and web-based tools [18,19], and to ensure relevance to current research and clinical contexts. A pilot search was first conducted in MEDLINE to confirm the sensitivity and accuracy of the strategy using four potentially relevant sentinel articles [12,20,21,22,23].

Search results were imported into Covidence, where duplicates were removed. Title and abstract screening were performed independently by two reviewers (A.K., K.L.) using predefined eligibility criteria, with discrepancies resolved through discussion. Full-text screening was completed independently by two reviewers (A.K., K.L.), with uncertainties adjudicated through consensus with the research team. Reference lists of included studies were manually screened to identify additional eligible publications.

Studies were included if they: (1) were observational research; (2) involved participants over two years of age with a medically diagnosed chronic condition (as defined by the criteria studies or recognised disease classifications, Supplementary Table S2); (3) reported completion rates for FRs or FFQs; and (4) were published on or after 2015. No restrictions were applied based on geographic location or study setting. Studies including healthy control groups were eligible, where a chronic condition group was also present. Exclusion criteria comprised studies that did not report completion rates, were not observational in design, or did not include populations with chronic conditions. Eligibility criteria are presented in Table 1.

**Table 1:** Eligibility Criteria.

Inclusion Criteria	Exclusion Criteria
Observational studies	Abstracts, conference proceedings, editorials, intervention studies.
Human Participants: Children aged 2–17 years or adults aged 18 years and older	Animal studies. Studies on the intake of infants less than two years.
Participants diagnosed with a chronic condition as per the MeSH definition, i.e., having one or more of the following characteristics: permanent residual disability that is caused by a non-reversible pathological alteration that requires rehabilitation, special training, and extended medical care and supervision of the patient [24] or healthy controls	Acute or temporary conditions.
Reported completion rates	Did not report on completion rates.
Published on or after 2015	Published before 2015.

Data extraction was completed independently by one author (A.K.) using a standardized data extraction form in Microsoft Excel (Version 16.102.1). A second reviewer (K.L. C.M, or G.P.) independently checked all extracted data for completeness. Any discrepancies were resolved through discussion, referring to the original articles when required. The data collected consisted of study demographics (author, year of publication, study location) and population characteristics (sample size, age, and gender ratio). Completion rates were extracted as reported in the original studies, including both numerator and denominator where available. Where sufficient data were provided, completion proportions were recalculated by the review authors as both a fraction and a percentage of the population. When not presented in this format, the percentage and/or fraction was calculated. Where outcome data were not available, it was recorded as ‘Not reported’ or ‘Unclear’ and no attempts were made to contact the authors of the study.

The Hoy Risk of Bias Tool for prevalence studies was used to assess the quality of the included studies [25]. This 10-item checklist was used to evaluate the methodological quality of the observational studies. The external validity was assessed by items 1 to 4, and the internal validity was assessed by items 5 to 10. Each item was rated “yes” (1) or “no” (0). A summary assessment was used to evaluate the overall risk of study bias following three categories: low risk (8–10), moderate risk (5–7), and high risk (0–4).

Basic descriptive statistics were used to describe the study population (age, gender, and chronic conditions). Meta-analyses were conducted using MedCalc^®^ Statistical Software (Version 23.3.7). Pooled completion rates were estimated using a random-effects model to account for anticipated heterogeneity across studies in terms of populations, study designs, and dietary assessment methods. Completion rates were meta-analysed according to type (FFQ vs. food record) or subtypes of FFQ or Food Record (e.g., 3-day vs. 7-day food records). Proportions were pooled to generate mean completion estimates with 95% confidence intervals. Heterogeneity between studies was assessed using the *I*^2^ statistic, with higher values indicating greater variability. Values of approximately 25%, 50%, and 75% were interpreted as indicating low, moderate, and high heterogeneity, respectively. Subgroup analyses were performed based on tool type, format, age group, disease category, and timing of assessment. Results are presented to an appropriate level of precision to avoid overinterpretation of estimates.

Publication bias was assessed using both Egger’s test [26] and visual inspection of funnel plots. Funnel plots were generated for overall analyses to evaluate potential asymmetry. However, both methods have limitations, particularly when the number of included studies is small, and interpretation was undertaken with caution. In line with methodological recommendations, publication bias assessments were considered unreliable where fewer than 10 studies were included in a given analysis. Statistical significance was set at *p* < 0.05.

Ethics approval was not required, as this systematic review utilised publicly available data. The results were reported according to the Preferred Reporting Items for Systematic Literature Reviews and Meta-Analyses (PRISMA) Guidelines [27].

## 3. Results

**Figure 1:**
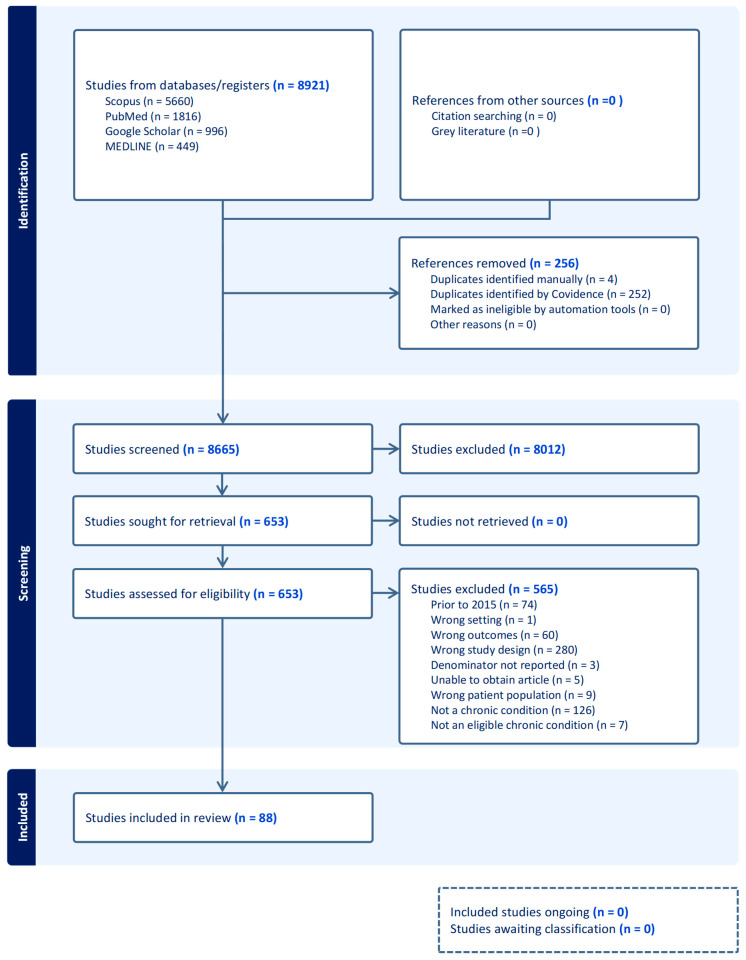
PRISMA Flow Diagram.

The initial database search resulted in 8921 articles, curated from Scopus (*n* = 5660), PubMed (*n* = 1816), Google Scholar Harzing’s Publish (*n* = 996) and Medline (*n* = 449). Following the removal of 256 duplicates (*n* = 252 identified through Covidence, *n* = 4 manually removed), 8665 studies were screened by title and abstract, of which 8012 were excluded. A total of 653 full-text articles were assessed for eligibility, with a further 565 studies excluded for various reasons such as wrong study design (*n* = 280), not a chronic condition/disease (*n* = 126) and (*n* = 74) studies published before 2015, as shown in Figure 1.

A total of 88 observational studies published in English between January 2015 and May 2025 were included in this review. Across the included studies, data were drawn from approximately 84,579 study samples, representing at least 79,182 unique participants. Supplementary Table S3 details the full characteristics of all included studies. The studies were derived from Europe (*n* = 31 [28,29,30,31,32,33,34,35,36,37,38,39,40,41,42,43,44,45,46,47,48,49,50,51,52,53,54,55,56,57,58,59]), North America (*n* = 29 [23,60,61,62,63,64,65,66,67,68,69,70,71,72,73,74,75,76,77,78,79,80,81,82,83,84,85,86]), Asia (*n* = 15 [87,88,89,90,91,92,93,94,95,96,97,98,99,100,101]), Australia/Oceania (*n* = 8 [102,103,104,105,106,107,108,109]), the Middle East (*n* = 4 [110,111,112,113]) and South America (*n* = 1 [114]), with no studies from Africa represented in the final review. Food Frequency Questionnaires accounted for most studies observed (*n* = 70 studies using 83 different FFQs, 79.5%), with the remaining derived from Food Records (*n* = 26, 29.5%). Across the 88 included studies, 70 examined adult populations, six included adults and children, and 12 were fully pediatric-focused. Adult samples ranged from fewer than 30 participants [45,83] to more than 13,000 participants [56]. In contrast, all pediatric samples were relatively small, and ranged from 38 children [60] to 785 children [106], with most 40–120 participants.

The conditions observed in the studies reviewed included Cancer (all types *n* = 29 (33%, [23,32,33,35,41,45,51,52,56,58,59,61,62,65,67,68,70,72,74,76,81,84,86,90,92,96,112,113,114]), Diabetes (Type 1, Type 2 or combined *n* = 17 (19.3%) [29,30,39,42,43,63,64,73,77,88,97,98,99,102,105,106,109]), Chronic Kidney Disease (*n* = 10, 11.4%, [28,31,37,80,87,91,93,94,101,103]), Gastrointestinal Diseases *n* = 14 (15.9%, [34,36,49,53,55,57,69,75,78,79,95,104,107,111]), Other (*n* = 6, [40,46,47,85,100,110]), Cardiometabolic Conditions *n* = 5 (5.6%, [48,50,54,71,89]), Neurological Conditions (*n* = 6, 6.8%, [38,44,60,66,82,83]), and Mental Illness (*n* = 1, [108]).

### 3.1. Completion Rates Overall

The overall pooled mean completion rate of the dietary assessment tools was 79.1% (95% Confidence Interval (CI): 74.37–83.43%, *n* = 84,579 participants, *n* = 88 studies) with estimates derived using random-effects meta-analysis, as shown in Table 2. High heterogeneity was observed amongst the studies with an *I*^2^ score of 99.68% and *p*-value of *p* < 0.0001, indicating high variability in completion outcomes across the data collection methods, study population and chronic condition types. Given the extremely high heterogeneity (*I*^2^ = 99.68%), the overall pooled estimate should be interpreted with caution and considered a summary measure rather than a precise estimate applicable across settings.

A summary of these key findings is visually presented in a funnel plot in Supplementary Figure S1. Interpretation of the funnel plot is limited due to high heterogeneity.

### 3.2. Completion Rates According to Tool Type (FFQ vs. FR)

The pooled mean proportion completion rate for Food Frequency Questionnaires was 80.6% (95% CI: 75.13–85.52%; 83 types). This completion rate differed between paper and electronic versions (paper 77.7%, 95% CI: 71.58–83.17%, 52 types and electronic 87.8%, 95% CI: 67.60–98.81%, 13 types, respectively, Table 2).

In contrast, the pooled mean proportion completion rate for Food Records was 74.3% (95% CI: 66.79–81.06%, Table 2, *n* = 28 types). Completion rates between paper and electronic forms were similar (paper 73.9%, 95% CI: 62.98–83.49%, and 22 types and electronic 75.0%, 95% CI: 64.42–84.30% and 6 types, respectively, Table 2).

### 3.3. Completion Rates According to Format

Length or duration of the tool appears to impact completion rates (Table 2). Short (1–100 item) FFQs had a completion rate of 81.2% (95% CI: 69.67–90.45%, *n* = 22 types), medium (101–150 items) FFQs had a rate of 80.7% (95% CI: 73.16–87.27%, *n* = 32 types) and long FFQs had a rate of (>151 items) 80.4% (95% CI: 67.37–90.71%, *n* = 24 types). This reduced further when separated according to paper or electronic format, with long paper FFQ completion rates reaching a pooled proportion of 74.3% (95% CI: 62.93–84.9%, *n* = 13 types).

Paper food records also had the lowest pooled proportion of 73.9% (95% CI: 62.98–83.49%, *n* = 12 types). Interestingly, short-duration food records (1 day) had the lowest pooled proportion (41.5%, 95% CI: 36.40–46.60%); however, this analysis consisted of only two studies.

### 3.4. Completion Rates According to Age

Completion rates for studies involving children were substantially lower than those for studies involving adults. For example, FFQ completion in children was estimated at 49.4% (95% CI: 36.14–62.70%, *n* = 4 types) compared to 81.9% in adults (95% CI: 76.43–86.78%, Table 2, *n* = 79 types). This was also the case for food records; however, the difference between ages was smaller: children 71.4% (95% CI: 43.04–92.71%, *n* = 6 types) compared to adults 75.0% (95% CI: 67.69–81.65%, *n* = 22 types). These estimates should be interpreted with caution due to the relatively small number of paediatric studies and the wide confidence intervals observed, indicating substantial uncertainty in the pooled estimates.

### 3.5. Completion Rates According to Disease Category

Studies of people with kidney disease had the highest mean pooled completion rates. This was the case for both FFQs (95.4%, 95% CI: 91.16–98.34%, *n* = 6 studies) and food records (76.1%, 95% CI: 56.923–90.90%, *n* = 6 studies, Table 2). Completion rates in studies of those with diabetes, cancer and gastrointestinal conditions varied according to format, with completion rates for FFQs higher than food records in all conditions (Table 2).

### 3.6. Completion Rates According to Timing of Assessment

Of the studies of FFQs, completion rates were lower at follow-up than at baseline. The mean pooled proportion at follow-up was 72.1% (95% CI: 61.82–81.25%, Table 2, *n* = 13 studies), compared to 82.1% at baseline (95% CI: 75.93–87.47%, *n* = 70 types). The completion rate at baseline for food records was 75.3% (95% CI: 67.72–82.08%, *n* = 27 types), with insufficient number of studies to be meta-analysed at follow-up.

### 3.7. Risk of Bias Assessment

Two authors independently assessed the risk of bias in 88 studies, as shown in Supplementary Table S4. Most studies demonstrated adequate internal validity based on checklist criteria. The overall score was 7.35 ± 1.35/10. Of these, 35 studies (39%) had a low risk of bias, and 53 had a moderate risk (59%). No study was considered to have a high risk of bias. Internal validity was rated sufficient in all studies for all items. However, seventy-three studies (83%) failed to report whether there were differences between responders and non-responders regarding completion of food intake tools [25,115]. Details about recruitment were also suboptimal, with most studies either being unclear or lacking in information regarding the random selection of participants.

## 4. Discussion

This systematic review and meta-analysis provide the first comprehensive synthesis of completion rates for Food Frequency Questionnaires (FFQs) and Food Records (FRs) used in observational studies involving people with chronic conditions. Drawing on data from more than 84,000 participants across 88 studies, several key findings are apparent: (i) FFQs achieved higher completion rates than FRs; (ii) electronic formats may be associated with higher completion rates than paper-based tools; (iii) adult studies reported higher completion than paediatric studies; (iv) completion rates varied by chronic condition; and (v) baseline assessments had higher completion than follow-up measures.

When considered at the subgroup level, these findings highlight the importance of respondent burden, delivery format, and population characteristics in the selection of dietary assessment tools for clinical and research settings. However, the extremely high heterogeneity observed across analyses substantially limits the interpretability of the overall pooled completion rate. This level of variability suggests that completion rates are highly context-specific and influenced by multiple interacting factors, rather than representing stable estimates across populations or tools. Consequently, subgroup findings provide more meaningful and actionable insights than the aggregate estimate. Overall, the results reflect associations observed across diverse study contexts and should not be interpreted as causal or prescriptive, underscoring the challenges of standardising dietary assessment methods in observational nutrition research. This also highlights the complexity of conducting observational nutritional research in order to answer important questions about health [116].

A key consideration when interpreting these findings is the substantial clinical heterogeneity of the populations included. This review synthesised data across a wide range of chronic conditions, including cardiometabolic diseases, cancer, neurological and psychiatric disorders, gastrointestinal conditions, and developmental disorders. These populations differ markedly in symptom burden, cognitive and functional capacity, caregiver involvement, treatment complexity, and exposure to dietary counselling. These differences are likely to influence the feasibility and acceptability of dietary assessment tools. As a result, pooled completion estimates may have limited clinical comparability and should be interpreted as broad indicators rather than directly transferable across specific patient groups.

Consistent with the longstanding methodological literature [117], FFQs demonstrated higher completion rates than food records. The relative efficiency of FFQs and their focus on habitual intake rather than day-to-day recording may contribute to higher acceptability, particularly in adults managing chronic disease. While food records are often favoured for their granular detail and reduced recall bias, their intensive nature appears to compromise feasibility, especially in large studies or populations with fluctuating symptoms, fatigue, or cognitive load. These findings suggest that, in many chronic disease contexts, the theoretical advantages of food records may be offset by lower completion and potential bias introduced by selective under-reporting.

The superior completion observed with electronic tools aligns with a growing body of evidence supporting digital dietary assessment. Features such as automated reminders, structured response fields, and mobile accessibility may reduce participant effort and facilitate integration into daily routines. Nevertheless, reliance on electronic formats must be balanced against issues of digital literacy, access, and preference. Paper-based tools may remain appropriate for some individuals, particularly older adults or those from culturally and linguistically diverse backgrounds, where annotation and interviewer support are valuable. A flexible, multimodal approach to dietary assessment is therefore essential to avoid exacerbating inequities in research participation.

Marked differences in completion between adult and paediatric populations were evident, particularly for FFQs. Lower completion rates in children likely reflect the cumulative burden placed on caregivers, challenges associated with proxy reporting, and the broader demands of managing chronic illness in family contexts. These findings highlight the need for age-appropriate, family-centred dietary assessment strategies, including simplified tools, visual supports, and integration with routine clinical care where possible. The relatively small difference observed for food records may reflect structured support in paediatric studies or shorter assessment periods, though the limited number of studies warrants cautious interpretation.

Completion rates also varied according to chronic condition, with higher rates observed in kidney disease cohorts [28,31,37,80,87,91,93,101,103] compared with diabetes, gastrointestinal, and oncological populations. This may reflect greater familiarity with dietary monitoring in conditions such as chronic kidney disease, where nutrition management is a core component of care and dietary education is often embedded within multidisciplinary services. However, these interpretations should be considered exploratory due to the limited number of studies and the potential confounding factors of the healthcare setting and study design. Conversely, lower completion in diabetes and gastrointestinal conditions may be influenced by complex dietary regimens, symptom variability, or symptom management fatigue [118]. These condition-specific patterns emphasise the importance of contextualising dietary assessment within disease trajectories and care models [119].

As anticipated, completion was higher at baseline than at follow-up, highlighting participant attrition and declining engagement over time. This has important implications for longitudinal studies, where repeated dietary assessments are common but may contribute substantially to cumulative study burden. Strategies to minimise the tool length, streamline follow-up assessments, and clearly communicate the value of ongoing participation are critical to maintaining data completeness.

A particularly important finding from the risk of bias assessment was that most studies (83%) did not report differences between responders and non-responders. This is an important finding and highly relevant given that the primary outcome of this review is completion of dietary assessment tools. Non-completion of tools is unlikely to occur at random and may be systematically associated with participant characteristics such as disease severity, symptom burden, cognitive fatigue, socioeconomic disadvantage, health literacy, and access to or familiarity with digital technologies. As a result, reported completion rates may overestimate the feasibility of dietary assessment tools if individuals who face greater barriers to participation are underrepresented. This introduces the potential for selective non-response bias and limits the interpretability and generalisability of the findings.

This review has several strengths, including the comprehensive search strategy, adherence to reporting guidelines and a large, diverse sample capturing a broad range of chronic conditions and age groups. However, several limitations should be considered when interpreting the findings.

First, the dominance of English-language studies may have led to the omission of relevant studies conducted in non-English-speaking contexts. In addition, there was an underrepresentation of studies from low- and middle-income countries, particularly from Africa, which limits the global generalisability of the findings.

A major limitation of the meta-analysis is the extremely high heterogeneity across studies. Although subgroup analyses were conducted to explore potential sources of variability, residual heterogeneity remained high, limiting the interpretability and generalisability of pooled estimates. This is further compounded by the inclusion of highly diverse chronic disease populations within a single analytical framework. Participants likely differed in terms of disease-related factors, cognitive and functional capacity, caregiver involvement (particularly in paediatric and developmental conditions), digital literacy, and exposure to dietary counselling. These factors are likely to influence completion rates and reduce clinical comparability across studies. While subgroup analyses by disease category and age group were undertaken, these may not fully account for within-category variability or contextual influences on participation. Furthermore, the influence of study quality on pooled estimates was not formally assessed through sensitivity or meta-regression analyses, which may limit insight into the robustness of findings.

Additional methodological limitations relate to inconsistencies in reporting across primary studies. While meta-analysis was feasible, the findings should be interpreted with caution when considering representativeness in the real world or broader populations living with chronic conditions. In particular, wide variation in the definition and reporting of completion rates, including inconsistent denominator use, limits comparability between studies and may introduce bias. Many studies also failed to report differences between responders and non-responders, raising the possibility of selective non-response bias, whereby completion rates may be overestimated if individuals facing greater participation barriers are underrepresented. Reporting of participant eligibility, recruitment, and completion was also inconsistent across studies, and many studies were excluded due to insufficient reporting of denominators or completion outcomes. Analyses were also conducted using the default proportion meta-analysis procedure within the software and no variance-stabilising transformation was applied. As a result, the influence of extreme proportions (e.g., values close to 0% or 100%) on pooled estimates cannot be excluded.

The inclusion of Google Scholar, while improving search sensitivity, introduces limitations in reproducibility due to its proprietary ranking algorithms. To mitigate this, a structured screening approach and predefined stopping rule were applied.

Finally, although risk of bias was assessed for all included studies, no sensitivity analyses were conducted to evaluate the influence of study quality on pooled estimates. No studies were classified as high risk using the Hoy Risk of Bias tool; therefore, exclusion-based analyses were not feasible. However, the potential impact of study quality and unmeasured bias on the findings cannot be excluded. Missing response bias reporting is a major limitation impacting the interpretability of completion rate findings.

From a practical perspective, these findings may be used by clinicians and researchers. Anticipated completion rates should be considered early in study design and clinical service planning, with selection of dietary assessment tools aligned to participant capacity, disease-related factors, and available support. Some evidence also suggests that completion on mobile devices for food intake is preferred over desktop or laptop forms, as it is less cognitively demanding and fits better into daily routines, with completion commonly occurring in the evening [120]. Therefore, short, electronically delivered FFQs, supported by clear instructions and visual aids, may offer advantages in terms of feasibility in some contexts. Person-centred approaches that prioritise accessibility, cultural relevance, and minimisation of study or completion burden are essential to optimise participation and data quality. Future research could explore the influence of study quality and methodological characteristics on completion rates using more detailed sensitivity or meta-regression approaches. Finally, improved standardisation in the reporting of completion rates, reporting of differences between responders and non-responders and participant flow would substantially enhance the interpretability and utility of future dietary assessment research.

## 5. Conclusions

This systematic review and meta-analysis highlight that completion rates of dietary assessment tools in populations living with chronic conditions vary substantially across populations, tool type, and study contexts. Short, electronically administered FFQs were associated with higher completion rates in the included studies, although this pattern was observed within specific study contexts and populations. While these pooled estimates provide a broad overview, the high heterogeneity observed indicates that these results should be cautiously interpreted and are not suggestive of inherent superiority. These findings support the need for multimodal person-centred tool selection and improved standardisation when reporting completion outcomes. Tailoring dietary assessment strategies to the characteristics and needs of specific patient groups remains essential to optimise data completeness and quality. However, tool selection should remain context-specific.

## Figures and Tables

**Table 2:** Completion rates meta-analysis summary table.

Group	Studies (*n*)	Participants (*n*)	Completion Rate (%)	95% Confidence Interval (%)	*I*^2^ (%)	*p* Value Heterogeneity
All	88	94,735	79.1	74.38–83.42	99.65	<0.0001
Food Frequency Questionnaires						
**Group**	**FFQ Types** **(*n*)**	**Participants** **(*n*)**	**Completion Rate** **(%)**	**95% Confidence Interval** **(%)**	***I* ^2^** **(%)**	***p* Value** **Heterogeneity**
All	83	86,229	80.6	75.13–85.52	99.72	<0.0001
Paper	52	45,289	77.7	71.58–83.17	99.52	<0.0001
Electronic	13	16,060	87.8	67.60–98.81	99.86	<0.0001
Both	2	1330	89.5	86.00–92.60	62.22	0.104
Unclear	15	23,550	81.9	69.30–91.68	99.72	<0.0001
Adults	79	84,363	81.9	76.43–86.78	99.73	<0.0001
Children	4	1866	49.4	36.14–62.70	96.47	0.0001
Cancer	33	44,992	77.4	70.93–83.19	99.53	<0.0001
Cardiometabolic	7	1527	88.8	82.60–93.79	91.63	0.0001
Diabetes	14	21,777	71.4	50.56–88.33	99.89	<0.0001
Gastrointestinal	10	3086	84.4	70.59–94.46	98.79	<0.0001
Kidney Disease	6	10,948	95.4	91.16–98.34	95.05	<0.0001
Mental Illness	-	-	-	-	-	-
Neurological	6	2732	80.2	69.38–89.11	97.13	<0.0001
Other	5	935	81.7	62.20–95.16	97.81	<0.0001
Baseline	70	72,226	82.1	75.93–87.47	99.75	<0.0001
Follow up	13	14,003	72.1	61.82–81.25	99.23	<0.0001
Short (1–100 items)	22	20,719	81.2	69.67–90.45	99.68	<0.0001
Medium (101–150)	32	39,903	80.8	73.16–87.27	99.67	<0.0001
Long (>151 items)	24	10,579	80.4	67.30–90.71	99.52	<0.0001
Short electronic	5	10,461	86.9	63.80–99.08	99.57	<0.0001
Medium electronic	2	1032	93.2	91.62–94.68	0.00	0.8669
Long electronic	5	3531	87.9	42.93–97.90	99.68	<0.0001
Short paper	14	7284	78.1	66.61–87.68	99.13	<0.0001
Medium paper	22	34,494	77.9	67.99–86.46	99.75	<0.0001
Long paper	13	2038	74.7	62.93–84.92	96.30	<0.0001
Food Records Only						
**Group**	**FR Types** **(*n*)**	**Participants** **(*n*)**	**Completion Rate** **(%)**	**95% Confidence Interval** **(%)**	***I*** **^2^** **(%)**	***p* Value** **Heterogeneity**
All	28	8506	74.3	66.79–81.06	98.16	<0.0001
Paper	22	4070	73.9	62.98–83.49	98.26	<0.0001
Electronic	6	4436	75.0	64.42–84.30	97.88	<0.0001
Adults	22	7053	75.0	67.69–81.65	97.62	<0.0001
Children	6	1453	71.4	43.04–92.71	99.14	<0.0001
Cancer	7	900	76.0	52.78–93.04	98.14	<0.0001
Diabetes	7	5033	67.6	52.490–80.93	99.07	<0.0001
Gastrointestinal	6	1440	68.1	57.33–77.95	94.11	<0.0001
Kidney Disease	6	1023	76.1	56.93–90.90	97.55	<0.0001
Baseline	27	8256	75.3	67.72–82.08	98.17	<0.0001
Short (1 day)	2	355	41.5	36.40–46.60	0.00	0.490
Medium (3 days)	20	7316	74.1	65.60–81.71	98.29	<0.0001
Long (7 days)	6	745	78.2	57.59–93.21	97.26	<0.0001

Legend: Cancer includes: Head and Neck; Bladder, Breast, Melanoma, Gastrointestinal, Colorectal, Oesophageal, Prostate, Stomach, Testicular, Ovarian, Glioma, Chemotherapy recipients; Cardiometabolic includes: Cardiovascular disease, Ischemic Heart Disease, Cardiac Rehabilitation; Gastrointestinal includes: Coeliac Disease, Inflammatory Bowel Disease; Irritable Bowel Syndrome: Pouchitis, Barrett’s Oesophagus, Dyspepsia; Other includes: Chronic Obstructive Pulmonary Disease, Rheumatoid Arthritis, HIV, Phenylketonuria; Neurological includes: Multiple Sclerosis, Parkinson’s Disease, Motor Neurone Disease, Autism Spectrum Disorder,. Kidney disease includes hemodialysis, peritoneal dialysis, kidney transplant, chronic kidney disease, nephrotic syndrome, gout.

## Data Availability

The original contributions presented in this study are included in the article/Supplementary Materials. Further inquiries can be directed to the corresponding author.

## Supplementary Materials

The following supporting information can be downloaded at https://www.mdpi.com/article/10.3390/nu18121922/s1. Supplementary Table S1: MEDLINE search strategy; Supplementary Table S2: Chronic conditions included in study; Supplementary Table S3: Characteristics of included studies (*n* = 88); Supplementary Table S4: Risk of Bias Assessment using Hoy assessment tool; Supplementary Figure S1: Funnel plot of all eligible studies.
