# Supplementary material for: Completion Rates of Food Frequency Questionnaires and Food Records in People with Chronic Conditions: Systematic Review and Meta-Analysis
Source: Nutrients. 2026 Jun 13;18(12):1922. doi: 10.3390/nu18121922 (PMC13306072; doi:10.3390/nu18121922)
Supplement: Supplementary file 1 [file nutrients-18-01922-s001.zip › supp Table S1 medline search terms proofed.pdf]

Supplementary Table S1. MEDLINE search strategy.

#	**Searches**
1	Diet History/
2	Questionnaires/
3	Self Report/
4	Diet Surveys/
5	Checklist/
6	(food adj3 (question* or survey* or record* or checklist*)).mp
7	Question*tw.
8	Survey*tw.
9	Checklist*.tw.
10	(screener* or screening).tw.
11	Ffq.mp.
12	Food frequency questionnaire or ffq
13	Food/
14	Food*
15	Diet/
16	Beverages/
17	Exp meals/
18	Water/
19	Meal*
20	Eat*
21	Snack*
22	Diet*
23	Intake/
24	((Diet or nutrition or food habit or eating habit or lifestyle or food))

25	Observational study/
26	Chronic Disease
27	(Review of literature or literature review or meta-analysis or systematic review)
28	S1 or S2 or S3 or S4 or S5 or S6 or S7 or S8 or S9 or S10 or S11 or S12
29	S13 or S14 or S15 or S16 or S17 or S18 or S19 or S20 or S21 or S22 or S23 or S24
30	S28 or S29
31	S25 or S28 or S29
32	S25 or S28 or S29 Limit Publication date to 01/01/2015-31/12/2025
33	S25 or S28 or S29 Human Studies
34	S26 or S33 or Observational Study
35	S26 and S33 Peer Reviewed
36	S34 Limit to English language
37	S27 and S36
