# Supplementary material for: Completion Rates of Food Frequency Questionnaires and Food Records in People with Chronic Conditions: Systematic Review and Meta-Analysis
Source: Nutrients. 2026 Jun 13;18(12):1922. doi: 10.3390/nu18121922 (PMC13306072; doi:10.3390/nu18121922)
Supplement: Supplementary file 1 [file nutrients-18-01922-s001.zip › supp Table S2 chronic conditions inlcuded revised for proof.pdf]

Supplementary Table S2: Chronic conditions included in study.

Chronic condition	Inclusions	Australian Prevalence (%)[a,b]
Cancer	Bowel or colorectal cancer, brain cancer, breast cancer, cancer of female genital organs, cancer of male genital organs, cancer of other digestive organs, cancer of other respiratory and intrathoracic organs, Hodgkin's disease, leukaemia, non-Hodgkin's lymphoma, oesophageal or stomach cancer, skin cancer, lung cancer, cancer site unknown, other malignant tumours, in situ neoplasms, benign neoplasms & neoplasms of uncertain nature	2.3
Cardiovascular diseases	Heart failure, ischaemic heart diseases (including angina, heart attack and other ischaemic heart diseases), stroke, other heart diseases, other heart stroke and vascular diseases (including other cerebrovascular diseases, oedema and diseases of arteries, arterioles and capillaries), other diseases of circulatory system (including rheumatic heart disease)	5.5
Chronic respiratory	Asbestosis, asthma, chronic obstructive pulmonary disease (chronic airflow limitation, chronic bronchitis, emphysema)	12.3
Endocrine disorders	Type 1 diabetes, Type 2 diabetes and diabetes type unknown	5.3
Gastrointestinal	Diseases of the liver, diseases of the oesophagus (including gastro oesophageal reflux disease), inflammatory bowel disease (enteritis and colitis)	1.7
Genitourinary	Chronic kidney disease (including Glomerular diseases, Renal failure or Kidney disease, Renal tubulo-interstitial diseases), non-inflammatory female pelvic conditions (including endometriosis)	2.7

Hearing and vision disorders[(c)]	Deafness or hearing loss (complete, partial, deaf mutism and other deafness or hearing loss nec.), other hearing and vestibular disorders (including, otosclerosis, Meniere's disease, tinnitus, other diseases of the middle ear and mastoid, other ear and mastoid)<br>Cataracts, glaucoma, macular degeneration, refractive errors (including long sight or hyperopia, short sight or myopia, astigmatism, presbyopia, other disorders of ocular muscles, binocular movement, accommodation and refraction) and other vision disorders (including complete and partial blindness, colour blindness, retinal disorders or defects, other diseases of the eye and adnexa)	21.3
Infectious diseases	AIDS (Auto Immune Deficiency Syndrome) or HIV (Human Immunodeficiency Virus), post COVID-19 conditions,	0.1
Infant and congenital conditions	Birth complications (including respiratory problems related to birth and other conditions originating in the perinatal period), cerebral palsy, congenital brain damage or malformation (including spina bifida), Down syndrome, other chromosomal abnormalities, other congenital conditions (including deformities of joints or limbs, other congenital malformations and deformations)	0.8
Mental and substance use	ADHD, anxiety disorders (including feeling anxious nervous or tense, obsessive compulsive disorder, panic attack, panic disorder, phobic anxiety disorders, post-traumatic stress disorder), autism spectrum disorders, bipolar affective disorder (including mania), conduct disorders, depression (including feeling depressed and other mood affective disorders), harmful use or dependence on alcohol, harmful use or dependence on drugs (including prescription drugs and other substances), intellectual impairment, schizophrenia (including psychosis), other mental health conditions (including dyslexia, dyslalia, speech impairment, other behavioural, cognitive and emotional problems with usual onset in childhood or adolescence and other mental and behavioural problems)	25.9
Musculoskeletal	Back problems (including sciatica, disc disorders and curvature of the spine), gout, osteoarthritis, rheumatoid arthritis, other musculoskeletal and connective tissue conditions (including acquired deformities of joints and limbs, soft tissue disorders, other arthritis and type unknown, other arthropathies, other diseases of the musculoskeletal system)	28.7
Neurological	Dementia (including Alzheimer's disease), epilepsy, migraine, motor neurone disease, multiple sclerosis, Parkinson's disease, other disease of the nervous system (including chronic fatigue	8.8

	syndrome, muscular dystrophy, narcolepsy, Huntington's disease)	
Skin	Dermatitis and eczema, psoriasis	4.1

a. Population prevalence estimates are based on information 'as reported' by NHS respondents and may differ from those reported from other sources due to differences in the method of data collection (for example, self-report survey compared with diagnostic survey).
b. The NHS is not conducted within institutions, such as residential aged care facilities, hospitals or prisons. This may exclude people likely to experience chronic conditions, leading to the underestimation of certain conditions, such as dementia.
c. Vision conditions corrected with glasses are excluded from analysis.

Data obtained from National Health Survey, Australia 2022 (Australian Bureau of Statistics)
