# Supplementary material for: Completion Rates of Food Frequency Questionnaires and Food Records in People with Chronic Conditions: Systematic Review and Meta-Analysis
Source: Nutrients. 2026 Jun 13;18(12):1922. doi: 10.3390/nu18121922 (PMC13306072; doi:10.3390/nu18121922)
Supplement: Supplementary file 1 [file nutrients-18-01922-s001.zip › supp Table S3 characteristics of included studies v2.pdf]

Supplementary Table S3. Characteristics of included studies (n=88).

Author	Location, Year	Type of Chronic Condition	Age Group/ Gender	Dietary Collection Tool (FFQ or FR), Paper or Electronic & Duration	Number of Participants	Completion Rate
Adanan et al., 2020 [1]	Malaysia, 2018	Chronic Kidney Disease (CKD)	Mean Age: 54.3 ± 12.2 years<br><br>Male: 55%	3-day Paper Food Record. (2 weekdays, 1 weekend day.)<br><br>Duration of Observation: 12 week follow up	Children: Nil<br><br>Adolescents: Nil<br><br>Adults:<br>B:68/87<br>P2: 68/87<br>P3:64/87<br><br>Total Participants: 87	Baseline FR:78.0%<br>Baseline FFQ: Nil<br><br>Point 2 FR:78.0%<br>Point 2 FFQ: Nil<br><br>Point 3 FR:74%<br>Point 3 FFQ: nil
Affret et al., 2017 [2]	France, 2017	Moderate to Advanced CKD	Mean Age: 65.3 years ± 11.8 years<br><br>Male: 64.7%	(1) 49-Item paper Short Food Frequency Questionnaire.<br><br>(2) 24-hour Food Record<br><br>Duration of Observation: 1 Year	Children: Nil<br><br>Adolescents: Nil<br><br>Adults:<br>(1) Baseline FFQ: 127/301<br>(2) Baseline FR: 244/301<br><br>Point 2 FR: Nil<br>Point 2 FFQ: 208/301<br><br>Point 3 FR: Nil<br>Point 3 FFQ: Nil<br><br>Total Participants: 301	Baseline FR: 42%<br>Baseline FFQ: 81%<br><br>Point 2 FR: Nil<br>Point 2 FFQ: 69%<br><br>Point 3 FR: Nil<br>Point 3 FFQ: Nil

Ahola et al., 2018 [3]	Finland, 2018	Type 1 Diabetes Mellitus	Mean Age: 47 years ± 14 years<br><br>Male: 45.5%	(1) 3-day App Food Record. (2 weekdays, 1 weekend day)<br><br>(2) 7 food group Food Frequency Questionnaire .<br><br>Duration of Observation: 2-years.	Children: Nil<br><br>Adolescents: Nil<br><br>Adults:<br>Baseline FR:1040/1429<br>Baseline FFQ:1429/1429<br><br>Total Participants: 1429	Baseline FR:73%<br>Baseline FFQ:100%<br><br>Point 2 FR: Nil<br>Point 2 FFQ: Nil<br><br>Point 3 FR: Nil<br>Point 3 FFQ: Nil
Ahola et al., 2021 [4]	Finland, 2021	Type 1 Diabetes Mellitus	Mean Age: 46 Years ± 14 Years<br><br>Male: 41.6%	3 Day Food Record (2 weekdays, 1 weekend day)<br><br>Duration of Observation 3 Months	Children: Nil<br><br>Adolescents: Nil<br><br>Adults:<br>Baseline FR: 1058/1867<br>Baseline FFQ: Nil<br><br>Point 2 FR: 689/1867<br>Point 2 FFQ: Nil<br><br>Total participants: 1867	Baseline FR: 56.7%<br>Baseline FFQ: Nil<br><br>Point 2 FR: 37%<br>Point 2 FFQ: Unable to ascertain<br><br>Point 3 FR: Nil<br>Point 3 FFQ: Nil
Amalia et al., 2019 [5]	United Kingdom, 2017	Chronic Kidney Disease	Mean Age: 62 Years ± 15.8 Years<br><br>Male: 57.8%	(1) Derby Salt Paper Food Frequency Questionnaire,<br><br>(2) Royal Free Sodium Questionnaire.<br><br>Duration of Observation: Unable to Ascertain	Children: Nil<br><br>Adolescents: Nil<br><br>Adults:<br>(1) DSQ:87/90<br>(2) RFSQ:86/90<br>Both 88/90<br><br>Total participants:90	Baseline DSQ:97%<br>Baseline RFSQ:96%<br>Baseline Both: 98%<br><br>Point 2 FR: Nil<br>Point 2 FFQ: Nil<br><br>Point 3 FR: Nil<br>Point 3 FFQ: Nil
Aponte et al., 2016 [6]	United States of America, 2016	Autism Spectrum Disorder	Mean Age: 6.9 years<br><br>Range 3.5 years - 12.5 years	167- Item Paper Food Frequency Questionnaire.<br><br>Duration of Observation: Unable to Ascertain	Children: 38<br><br>Adolescents: Nil<br><br>Adults: Nil	Baseline FR: Nil<br>Baseline FFQ:70.3%<br><br>Point 2 FR: Nil<br>Point 2 FFQ: Nil

			Male: 84.2%		Total participants: 54	Point 3 FR: Nil<br>Point 3 FFQ: Nil
Arthur et., 2018 [7]	United States of America, 2018	Head and Neck Cancer (Squamous Cell Carcinoma)	Mean Age 60.9 years ± 11 Years<br><br>Male: 76%	131- item paper self-administered Harvard Food Frequency Questionnaire<br><br>Duration of Observation: 5 years	Children: Nil<br><br>Adolescents: Nil<br><br>Adults:<br>Baseline FR: Nil<br>Baseline FFQ:440/520<br><br>Point 2 FR: Nil<br>Point 2 FFQ: 303/520<br><br>Point 3 FR:<br>Point 3 FFQ: 414/520<br><br>Total Participants: 520	Baseline FR: Nil<br>Baseline FFQ:84.6%<br><br>Point 2 FR:<br>Point 2 FFQ: 58.3%<br><br>Point 3 FR: Nil<br>Point 3 FFQ:80%
Bail et al., 2022 [8]	United States of America, 2021	Metastatic Cancer	Mean Age: 61.5 Years ± 12.95<br><br>Male: 49%	10 -Item Paper Food Frequency Questionnaire<br><br>Duration of Observation: Unable to Ascertain	Children: Nil<br><br>Adolescents: Nil<br><br>Adults:277/542<br><br>Total Participants: 277	Baseline FR: Nil<br>Baseline FFQ: 51%<br><br>Point 2 FR: Nil<br>Point 2 FFQ: Nil<br><br>Point 3 FR: Nil<br>Point 3 FFQ: Nil
Baleato et al., 2022 [9]	Australia, 2022	Diabetes Mellitus	Mean Age: 64.3 years<br><br>Male: Unable to Ascertain	74 food and beverage dietary questionnaire (Unclear Format)<br><br>Duration of Observation: 5 years	Children: Nil<br><br>Adolescents: Nil<br><br>Adults: 9102/9151<br><br>Total Participants: 9151	Baseline FR:<br>Baseline FFQ: 99.5%<br>Point 2 FR: Nil<br>Point 2 FFQ: Nil<br><br>Point 3 FR: Nil<br>Point 3 FFQ: Nil

Basu et al., 2019 [10]	United States of America, 2019	Type 1 Diabetes vs Control	Mean Age:39.0 years ± 9.0 (Both Groups)<br><br>Male: 47%	126 Item Paper Food Frequency Questionnaire<br><br>Duration of Observation: 2 Years	Children: Nil<br><br>Adolescents: Nil<br><br>Adults:<br>T1DM: 568/1257<br>Control: 689/1257<br><br>Baseline FFQ Total: 1257/1416<br><br>Total Participants: 1416	Baseline FR: Nil<br>Baseline FFQ:89.0%<br><br>Point 2 FR: Nil<br>Point 2 FFQ: Nil<br><br>Point 3 FR: Nil<br>Point 3 FFQ: Nil
Basu et al., 2021 [11]	United States of America, 2019	Type 1 Diabetes vs Control	Mean Age:38.0 years ± 9.0 (Both Groups)<br><br>Male: 47%	126 Item Paper Food Frequency Questionnaire<br><br>Duration of Observation: Unable to Ascertain	Children: Nil<br><br>Adolescents: Nil<br><br>Adults:<br>Baseline FR: Nil<br>Baseline FFQ:<br>T1D: 568/1257<br>Control: 689/1257<br><br>Point 2 FR: Nil<br>Point 2 FFQ:<br>T1D: 452/1257<br>Control: 538/1257<br><br>Total Participants: 1257	Baseline FR Nil<br>Baseline FFQ:<br>T1D: 45.2%<br>Control: 55%<br><br>Point 2 FR: Nil<br>Point 2 FFQ:<br>T1DM: 36%<br>Control: 43%<br><br>Point 3 FR: Nil<br>Point 3 FFQ: Nil
Beeren et al., 2021 [12]	Netherlands,2021	Bladder Cancer	Mean Age: 66.2 years<br><br>Male: 80%	163 Item Paper and Electronic Food Frequency Questionnaire<br><br>Duration of Observation: 6 weeks, 3 months and 15 months	Children: Nil<br><br>Adolescents: Nil<br><br>Adults: 949/1076<br><br>Total Participants: 1076	Baseline FR: Nil<br>Baseline FFQ:88.2%<br><br>Point 2 FR: Nil<br>Point 2 FFQ: Nil<br><br>Point 3 FR: Nil<br>Point 3 FFQ: Nil

Beiner et al., 2023 [13]	United States of America, 2023	Breast Cancer	Mean Age: Unable to Ascertain<br><br>Male: 0%	12-Item Electronic Food Frequency Questionnaire<br><br>Duration of Observation: Unable to Ascertain	Children: Nil<br><br>Adolescents: Nil<br>Adults:<br>P1:169/169<br>P2:160/169<br><br>Total Participants: 160	Baseline FR:<br>Baseline FFQ:100%<br><br>Point 2 FR: Nil<br>Point 2 FFQ: 94.4%<br><br>Point 3 FR: Nil<br>Point 3 FFQ: Nil
Belle et al., 2021 [14]	Switzerland,2021	Childhood Cancer Survivors	Mean Age: Unable to Ascertain<br><br>Median Age: 34 years<br><br>Male: 50%	97 Item Paper self-administered, semi-quantitative Bus Santé (FFQ) and Co Laus surveys.<br><br>Duration of Observation: Unable to Ascertain	Children: Nil<br><br>Adolescents: Nil<br><br>Adults:<br>Bus Santé:<br>2682/2770<br>Co Laus survey:<br>1322 /1448<br><br>Total Participants: 4218	Baseline FR: Nil<br>Baseline FFQ:<br>Bus Santé: 97.0%<br>Co Laus: 92.0%<br><br>Point 2 FR: Nil<br>Point 2 FFQ: Nil<br><br>Point 3 FR: Nil<br>Point 3 FFQ: Nil
Birketvedt et al., 2020 [15]	Norway,<br>2020	Oesophageal Atresia	Mean Age:16 Years<br><br>Male: 59%	4-Day Paper Dietary Record<br>(3 weekdays and 1 weekend)<br><br>Duration of Observation: Unclear	Children: Nil<br><br>Adolescents:<br>P1: 68/102<br>P2: 48/102<br><br>Adults: Nil<br><br>Total Participants:102	Baseline FR:67.0%<br>Baseline FFQ: Nil<br><br>Point 2 FR:47.1%<br>Point 2 FFQ: Nil<br><br>Point 3 FR: Nil<br>Point 3 FFQ: Nil
Black et al., 2021 [16]	United States of America ,2021	Multiple Sclerosis and Control	Mean Age: Unable to Ascertain<br><br>Male: Unable to Ascertain	13 Food category- Self-administered Paper Questionnaires<br><br>Duration of Observation: Unable to Ascertain	Children: Nil<br><br>Adolescents: Nil<br><br>Adults: 1061/1255<br><br>Total Participants: 1255	Baseline FR: Nil<br>Baseline FFQ:85%<br><br>Point 2 FR: Nil<br>Point 2 FFQ: Nil

						Point 3 FR: Nil<br>Point 3 FFQ: Nil
Bolte et al., 2023 [17]	Netherlands, 2023	Advanced Melanoma	Mean Age (Dutch): 59.3 ±12.74 Years<br><br>Mean Age (English): 66.21 ± 16.63 Years<br><br>Male: 50%	Epic-Norfolk Food Frequency Questionnaire<br><br>Dutch Healthy Diet (DHD) Food Frequency Questionnaire (Unclear Format)<br><br>Duration of Observation: Unable to Ascertain	Children: Nil<br><br>Adolescents: Nil<br><br>Adults: Participants<br>(1) Dutch 44/91<br>(2) British 47/91<br><br>Total Participants: 91	Baseline FR: Nil<br>Baseline FFQ:<br>(1) 48.4%<br>(2) 52.0%<br><br>Point 2 FR: Nil<br>Point 2 FFQ: Nil<br><br>Point 3 FR: Nil<br>Point 3 FFQ: Nil
Boucher et al., 2018 [18]	Canada, 2017	Breast Cancer	Mean Age:56 years<br><br>Male: 0%	20-Item Self-Administered Paper Food Frequency Questionnaire<br><br>Duration of Observation:2 Months	Children: Nil<br><br>Adolescents: Nil<br><br>Adults:<br>B:278/417<br><br>Total Participants: 417	Baseline FR: Nil<br>Baseline FFQ: 67%<br><br>Point 2 FR: Nil<br>Point 2 FFQ: Nil<br><br>Point 3 FR: Nil<br>Point 3 FFQ: Nil
Bredin et al., 2020 [19]	Ireland, 2017	Non-Alcoholic Fatty Liver Disease (NAFLD), and Non-Alcoholic Steatohepatitis (NASH)	Mean Age:58 Years<br><br>Male: 53%	(1) 48-Item Paper Short Food Frequency Questionnaire<br><br>(2) 4 Day Food Diary. 2 weekdays and 2 weekend days.<br><br>Duration of Observation:30 weeks	Children: Nil<br><br>Adolescents: Nil<br><br>Adults: 55/81<br><br>Total Participants: 81	Baseline FR:68%<br>Baseline FFQ:68%<br><br>Point 2 FR: Nil<br>Point 2 FFQ: Nil<br><br>Point 3 FR: Nil<br>Point 3 FFQ: Nil
Chhabra et al., 2024 [20]	United Kingdom, 2021	Chronic Kidney Disease	Mean Age: 63.8 ±16.1 Years<br><br>Male: 63%	Paper Sodium Food Frequency Questionnaire<br><br>Duration of Observation: Unable to Ascertain	Children: Nil<br><br>Adolescents: Nil<br><br>Adults:<br>111/115	Baseline FR: Nil<br>Baseline FFQ:96.5%<br><br>Point 2 FR: Nil<br>Point 2 FFQ: Nil

					Total Participants: 115	Point 3 FR: Nil<br>Point 3 FFQ: Nil
Coe et al., 2020 [21]	United Kingdom, 2020	Parkinsons Disease	Mean Age: 68 ± 9.64 Years<br><br>Male: 58%	EPIC-Norfolk Paper Food Frequency Questionnaire<br><br>Duration of Observation: 12 months	Children: Nil<br><br>Adolescents: Nil<br><br>Adults:<br>Baseline FFQ: 121/200<br>Point 2 FFQ: 90/200<br><br>Total Participants: 200	Baseline FR: Nil<br>Baseline FFQ:61%<br><br>Point 2 FR: Nil<br>Point 2 FFQ:45%<br><br>Point 3 FR: Nil<br>Point 3 FFQ: Nil
Conley et al., 2022 [22]	Australia, 2022	Chronic Kidney Disease	Mean Age: 64 ± 12 .4 years<br><br>Male: 68 %	7 Day self-administered Electronic Diet Record<br><br>Duration of Observation: Unable to Ascertain	Children: Nil<br><br>Adolescents: Nil<br><br>Adults:<br>90/119<br><br>Total Participants: 119	Baseline FR:75.6%<br>Baseline FFQ: Nil<br><br>Point 2 FR: Nil<br>Point 2 FFQ: Nil<br><br>Point 3 FR: Nil<br>Point 3 FFQ: Nil
Cooke et al., 2023 [23]	Australia, 2023	Dyspepsia	Mean Age: 32.5 years<br><br>Male 65%	297-point Electronic Semiqualitative Food frequency questionnaire.<br><br>Duration of Observation: 2 years	Children: Nil<br><br>Adolescents: Nil<br><br>Adults: 173/179<br><br>Total Participants: 179	Baseline FR: Nil<br>Baseline FFQ: 97.0%<br><br>Point 2 FR: Nil<br>Point 2 FFQ: Nil<br><br>Point 3 FR: Nil<br>Point 3 FFQ: Nil
Crowder et al., 2021 [24]	United States of America, 2021	Head and Neck Cancer Survivors	Mean Age: 62.7 ± 11.8 years<br><br>Male: 59.5 %	131-item paper self-administered Harvard Food Frequency Questionnaires<br><br>Duration of Observation: Unclear	Children: Nil<br><br>Adolescents: Nil<br><br>Adults: 42/79<br><br>Total Participants: 79	Baseline FR: Nil<br>Baseline FFQ: 53%<br><br>Point 2 FR: Nil<br>Point 2 FFQ: Nil<br><br>Point 3 FR: Nil<br>Point 3 FFQ: Nil

Dewinter et al., 2015 [25]	Belgium, 2015	Type 1 Diabetes Mellitus	Mean Age: Unable to Ascertain<br><br>Male: % Unable to Ascertain	7 Food Category Electronic Food Frequency Questionnaire<br><br>Duration of Observation: Unclear	Children: 44/98<br><br>Adolescents: 59/144<br><br>Total Children/Adolescents: 103/242<br><br>Adults: Nil<br><br>Total Participants: 242	Baseline FR: Nil<br>Baseline FFQ: 43.0%<br><br>Point 2 FR: Nil<br>Point 2 FFQ: Nil<br><br>Point 3 FR: Nil<br>Point 3 FFQ: Nil
Dinparast et al., 2021 [26]	Iran, 2021	Chronic Obstructive Pulmonary Disease (COPD) and Depression	Mean Age: 54.58 ± 5.08 Years<br><br>Male: 56.6%	132 item Paper Food Frequency Questionnaire<br><br>Duration of Observation: Unclear	Children: Nil<br><br>Adolescents: Nil<br><br>Adults: 220/220<br><br>Total Participants: 220	Baseline FR: Nil<br>Baseline FFQ: 100%<br><br>Point 2 FR: Nil<br>Point 2 FFQ: Nil<br><br>Point 3 FR: Nil<br>Point 3 FFQ: Nil
Dolovich et al., 2022 [27]	Canada, 2022	Irritable Bowel Disease	Mean Age: Unable to Ascertain<br><br>Male: % Unable to Ascertain	131-item Electronic Self-administered Harvard Food Frequency Questionnaire<br><br>Duration of Observation: Unclear	Children: Nil<br><br>Adolescents: Nil<br><br>Adults: 153/153<br><br>Total Participants: 153	Baseline FR: Nil<br>Baseline FFQ:100%<br><br>Point 2 FR: Nil<br>Point 2 FFQ: Nil<br><br>Point 3 FR: Nil<br>Point 3 FFQ: Nil
Dratsky et al., 2024 [28]	United States of America, 2024	Gastrointestinal Cancers	Mean Age: 63 ± 13 Years<br><br>Male: 42%	Electronic Food Frequency Questionnaire (Vio Screen ™)<br><br>Duration of Observation: Unclear	Children: Nil<br><br>Adolescents: Nil<br><br>Adults: 40/40<br><br>Total Participants: 40	Baseline FR: Nil<br>Baseline FFQ: 100%<br><br>Point 2 FR: Nil<br>Point 2 FFQ: Nil<br><br>Point 3 FR: Nil<br>Point 3 FFQ: Nil

Drzymała-Czyz et al., 2018 [29]	Poland, 2018	Phenylketonuria (PKU)	Mean Age: 21.3 years<br><br>Male: 50 %	3-day diary (Two weekdays and one weekend day)<br><br>Duration of Observation: 3 days	Children: 12/80<br><br>Adolescents: Nil<br><br>Adults: 68/80<br><br>Total Participants: 80	Baseline FR: Nil<br>Baseline FFQ:100%<br><br>Point 2 FR: Nil<br>Point 2 FFQ: Nil<br><br>Point 3 FR: Nil<br>Point 3 FFQ: Nil
Ericson et al., 2020 [30]	Sweden, 2020	Oesophageal Cancer	Median Age: 66 years<br><br>Male: 80%	24 Hr Paper Food Record<br><br>Duration of Observation: 24 hours	Children: Nil<br><br>Adolescents: Nil<br><br>Adults: 20/54<br><br>Total Participants: 54	Baseline FR: Nil<br>Baseline FFQ: 37%<br><br>Point 2 FR: Nil<br>Point 2 FFQ: Nil<br><br>Point 3 FR: Nil<br>Point 3 FFQ: Nil
Ewers et al., 2019 [31]	Denmark, 2019	Type 1 and Type 2 Diabetes	Mean Age: 48 Years<br><br>Male: 48 %	270 food items and mixed dishes web-based semi-quantitative food frequency questionnaire.<br><br>Duration of Observation: 3 months.	Children: Nil<br><br>Adolescents: Nil<br><br>Adults:774/3000<br>T1D: 426<br>T2D: 348<br><br>Total Participants: 3000	Baseline FR: Nil<br>Baseline FFQ: 26%<br><br>Point 2 FR: Nil<br>Point 2 FFQ: Nil<br><br>Point 3 FR: Nil<br>Point 3 FFQ: Nil
Ferrari et al., 2015 [32]	Brazil, 2015	Colorectal Cancer	Median Age: 61 years<br><br>Male: 51.79%	110 Item Paper Food Frequency Questionnaire<br><br>Duration of Observation: Unable to Ascertain	Children: Nil<br><br>Adolescents: Nil<br><br>Adults: 189/195<br><br>Total Participants: 195	Baseline FR: Nil<br>Baseline FFQ: 97.0%<br><br>Point 2 FR: Nil<br>Point 2 FFQ: Nil<br><br>Point 3 FR: Nil<br>Point 3 FFQ: Nil
Fisher et al., 2023 [33]	Australia, 2023	Type 1 Diabetes	Mean Age: 11.7 ± 2.9 Years	3-day weighed Paper Food records (2 weekdays and 1 weekend day)	Children: 21/48<br><br>Adolescents: 27/48	Baseline FR: 15.1 %<br>Baseline FFQ: Nil

			Male: 63%	Duration of Observation: 2 Years	Total Children and Adolescents: 48/318<br><br>Adults: Nil<br><br>Total Participants: 318	Point 2 FR: Nil<br>Point 2 FFQ: Nil<br><br>Point 3 FR: Nil<br>Point 3 FFQ: Nil
Ganguzza et al., 2018 [34]	United States of America, 2018	Cardiovascular Disease	Mean Age: 62 years<br><br>Male: 56.3 %	24- Item Paper Rate Your Plate (RYP) Food Frequency Questionnaire.<br><br>Duration of Observation: Unable to Ascertain	Children: Nil<br><br>Adolescents: Nil<br><br>Adults: 400/478<br><br>Total Participants: 478	Baseline FR: Nil<br>Baseline FFQ: 84%<br><br>Point 2 FR: Nil<br>Point 2 FFQ: Nil<br><br>Point 3 FR: Nil<br>Point 3 FFQ: Nil
Gilbertson et al., 2018 [35]	Australia, 2018	Type 1 Diabetes	Mean Age: Unable to Ascertain<br><br>Male: 50.6 %	(1) 120-item paper Australian Child and Adolescent Eating Survey Food Frequency Questionnaire (ACAES-FFQ)<br><br>(2) 17-item paper Food Frequency questionnaire<br><br>Duration of Observation: Unclear	Children:<br>FFQ (1) 429/785<br>FFQ (2) 266/785<br><br>Adolescents: Inclusive of child total.<br><br>Adults: Nil<br><br>Total Participants: 785	Baseline FR: Nil<br>Baseline FFQ(1): 55%<br>Baseline FFQ (2): 34%<br><br>Point 2 FR: Nil<br>Point 2 FFQ: Nil<br><br>Point 3 FR: Nil<br>Point 3 FFQ: Nil
Gingras et al., 2015 [36]	Canada, 2015	Type 1 Diabetes Mellitus	Mean Age: 44.3 ± 12.3 Years<br><br>Male:48.3 %	3-day Electronic Food Record<br><br>Duration of Observation: Unclear	Children: Nil<br><br>Adolescents: Nil<br><br>Adults: 118/124<br><br>Total Participants: 124	Baseline FR: 95.2 %<br>Baseline FFQ: Nil<br><br>Point 2 FR: Nil<br>Point 2 FFQ: Nil<br><br>Point 3 FR: Nil<br>Point 3 FFQ: Nil

Godny et al., 2019 [37]	Isreal, 2019	Pouchitis	Mean Age: 44.9 ± 14 years<br><br>Male: 41%	106-Item Food Frequency Questionnaire (Unclear Format)<br><br>Duration of Observation: 5 years	Children: Nil<br><br>Adolescents: Nil<br><br>Adults:<br>P1:172/172<br>P2:108/172<br>P3: 58/172<br>P4: 20/172<br><br>Total Participants: 172	Baseline FR: Nil<br>Baseline FFQ: 100%<br><br>Point 2 FR: Nil<br>Point 2 FFQ: 63%<br><br>Point 3 FR: Nil<br>Point 3 FFQ: 34%<br><br>Point 4 FR: Nil<br>Point 4 FFQ: 12%
Gregg et al., 2019 [38]	United States of America, 2019	Prostate Cancer	Mean Age: 64.4 Years<br><br>Male: 100 %	170-item Food Frequency Questionnaire (Unclear Format)<br><br>Duration of Observation: 6 Years	Children: Nil<br><br>Adolescents: Nil<br><br>Adults:<br>B: 501/560<br>6 mths: 263/411<br>36 mths: 76/411<br><br>Total Participants: 560	Baseline FR: Nil<br>Baseline FFQ: 89.5%<br><br>Point 2 FR: Nil<br>Point 2 FFQ: 64.0%<br><br>Point 3 FR: Nil<br>Point 3 FFQ:18.5%
Grieco et al., 2022 [39]	United States of America, 2022	Female Cancer Survivors	Mean Age: 73.6 ± 6.1 Years<br><br>Male: 0%	36-Item Paper, Electronic and Phone Food Frequency Questionnaire<br><br>Duration of Observation: Unclear	Children: Nil<br><br>Adolescents: Nil<br><br>Adults: 173/215<br><br>Total Participants: 215	Baseline FR: Nil<br>Baseline FFQ: 80.4%<br><br>Point 2 FR: Nil<br>Point 2 FFQ: Nil<br><br>Point 3 FR: Nil<br>Point 3 FFQ: Nil
Helm et al., 2024. [40]	United States of America, 2024	Type 1 Diabetes Mellitus	Mean Age: 39 ± 9 Years<br><br>Male: 50%	126-Item Food Frequency Questionnaire. (Unclear Format)<br><br>Duration of Observation: 3 years	Children: Nil<br><br>Adolescents: Nil<br><br>Adults:<br>B:563/652<br>P2: 411/652	Baseline FR: Nil<br>Baseline FFQ: 86.3%<br><br>Point 2 FR: Nil<br>Point 2 FFQ: 68%<br><br>Point 3 FR: Nil

					Total Participants: 652	Point 3 FFQ: Nil
Horikawa et al., 2022 [41]	Japan, 2022	Type 2 Diabetes Mellitus	Mean Age:<br>61.5 ± 7.8 years<br><br>Male: 60.2 %	58-Item Food Frequency Questionnaire.<br>(Unclear Format)<br><br>Duration of Observation:<br>Unable to Ascertain	Children: Nil<br><br>Adolescents: Nil<br><br>Adults: 1643/1992<br><br>Total Participants: 1992	Baseline FR: Nil<br>Baseline FFQ: 82.5%<br><br>Point 2 FR: Nil<br>Point 2 FFQ: Nil<br><br>Point 3 FR: Nil<br>Point 3 FFQ: Nil
Huisman et al., 2015 [42]	Netherlands, 2015	Motor Neuron Disease (MND)	Mean Age: 63.6 ± 11.0 years<br><br>Male: 62.0%	199- Item Paper Food Frequency Questionnaire<br><br>Duration of Observation:<br>1 year.	Children: Nil<br><br>Adolescents: Nil<br><br>Adults: 747/885<br><br>Total Participants: 885	Baseline FR:<br>Baseline FFQ:84.4%<br><br>Point 2 FR: Nil<br>Point 2 FFQ: Nil<br><br>Point 3 FR: Nil<br>Point 3 FFQ: Nil
Hu et al., 2015 [43]	Canada, 2015	Stomach Cancer	Mean Age: 61.9 ± 9.8 years<br><br>Male: 68.0%	69-Item Paper Food Frequency Questionnaire<br><br>Duration of Observation:<br>Unclear	Children: Nil<br><br>Adolescents: Nil<br><br>Adults: 1181/1809<br><br>Total Participants: 1809	Baseline FR: Nil<br>Baseline FFQ: 65.3%<br><br>Point 2 FR: Nil<br>Point 2 FFQ: Nil<br><br>Point 3 FR: Nil<br>Point 3 FFQ: Nil
Hussain et al., 2020 [44]	United States of America, 2020	Liver Cirrhosis	Mean Age: 57 years<br><br>Median Age:<br><br>Male:57 %	Electronic Photo Food Frequency Questionnaire<br>Vio Screen<br><br>Duration of Observation:<br>Unclear	Children: Nil<br><br>Adolescents: Nil<br><br>Adults: 51/165<br><br>Total Participants: 165	Baseline FR: Nil<br>Baseline FFQ: 31%<br><br>Point 2 FR: Nil<br>Point 2 FFQ: Nil<br><br>Point 3 FR: Nil<br>Point 3 FFQ: Nil
Ijpma et al., 2017 [45]	Netherlands, 2017	Testicular Cancer	Mean Age: 32 years	183-Item Paper Food Frequency Questionnaire	Children: Nil	Baseline FR: Nil<br>Baseline FFQ:95.2%

			Male:100 %	Duration of Observation: Unable to Ascertain	Adolescents: Nil<br><br>Adults:<br>B: 20/21<br>P2 Follow up:18/21<br>P3:18/21<br>P4: 15/21<br>P5:9/21<br>P6: 7/21<br><br>Total Participants: 21	Point 2 FR: Nil<br>Point 2 FFQ:86.0%<br><br>Point 3 FR: Nil<br>Point 3 FFQ: 86.0%<br><br>Point 4 FR: Nil<br>Point 4 FFQ: 71.4%<br><br>Point 5 FR: Nil<br>Point 5 FFQ: 43.0%<br><br>Point 6 FR: Nil<br>Point 6 FFQ: 33.33%
Ilari et al., 2021 [46]	Italy, 2021	COPD	Mean Age: 72.8 ± 8.8 years<br><br>Male:44.3 %	Paper Food Frequency Questionnaire<br>(Item value unknown)<br><br>Duration of Observation: 3 weeks	Children: Nil<br><br>Adolescents: Nil<br><br>Adults: 80/105<br><br>Total Participants: 105	Baseline FR: Nil<br>Baseline FFQ:76.2%<br><br>Point 2 FR: Nil<br>Point 2 FFQ: Nil<br><br>Point 3 FR: Nil<br>Point 3 FFQ: Nil
Khatun et al., 2021 [47]	Bangladesh, 2021	Coronary Artery Disease<br>(CAD)	Mean Age:<br>51.5 ± 9.0 years<br><br>Male: 53%	57- Item Paper Food Frequency Questionnaire<br><br>Duration of Observation: Unclear	Children: Nil<br><br>Adolescents: Nil<br><br>Adults: 100/105<br><br>Total Participants: 105	Baseline FR: Nil<br>Baseline FFQ: 95.2%<br><br>Point 2 FR: Nil<br>Point 2 FFQ: Nil<br><br>Point 3 FR: Nil<br>Point 3 FFQ: Nil
Kiew et al., 2022 [48]	Malaysia, 2022	Breast Cancer	Mean Age: Unable to Ascertain<br><br>Male: 0%	3-day Paper Food Record	Children: Nil<br><br>Adolescents: Nil<br><br>Adults:	Baseline FR:<br>(1) 69.2 %<br>(2) 44.8<br>Baseline FFQ: Nil

				Duration of Observation: Unclear	(1) 173/250<br>(2) 112/250<br><br>Total Participants: 250	Point 2 FR: Nil<br>Point 2 FFQ: Nil<br><br>Point 3 FR: Nil<br>Point 3 FFQ: Nil
Klimek et al., 2020 [49]	Germany, 2020	Phenylketonuria (PKU)	Mean Age: 33 ± 8.5 years<br><br>Male: 34.0%	91-Item Electronic Food Frequency Questionnaire<br><br>Duration of Observation: Unable to Ascertain	Children: Nil<br><br>Adolescents: Nil<br><br>Adults: 144/183<br><br>Total Participants: 183	Baseline FR: Nil<br>Baseline FFQ: 78.7%<br><br>Point 2 FR: Nil<br>Point 2 FFQ: Nil<br><br>Point 3 FR: Nil<br>Point 3 FFQ: Nil
Knoerl et al., 2024 [50]	United States of America, 2024	Cancer Survivors	Mean Age: 54.17 ± 11.93 years<br><br>Male: 7%	Electronic (Vio Screen) 13 Food group Food Frequency Questionnaire<br><br>Duration of Observation: Unable to Ascertain	Children: Nil<br><br>Adolescents: Nil<br><br>Adults: 140/143<br><br>Total Participants: 143	Baseline FR: Nil<br>Baseline FFQ: 98.0%<br><br>Point 2 FR: Nil<br>Point 2 FFQ: Nil<br><br>Point 3 FR: Nil<br>Point 3 FFQ: Nil
Kristensen et al., 2024 [51]	Denmark, 2024	Ischaemic Heart Disease (IHD)	Mean Age: Unable to Ascertain<br><br>Median Age: 64 years<br><br>Male: 76%	19- Item Paper Food Frequency Questionnaire<br><br>Duration of Observation: Unable to Ascertain	Children: Nil<br><br>Adolescents: Nil<br><br>Adults:<br>B:157/223<br>P2: 151/223<br><br>Total Participants: 223	Baseline FR: Nil<br>Baseline FFQ:70.4%<br><br>Point 2 FR: Nil<br>Point 2 FFQ: 68.0%<br><br>Point 3 FR: Nil<br>Point 3 FFQ: Nil
Lang et al., 2021 [52]	Germany, 2021	Non-Alcoholic Fatty Liver Disease (NAFLD)	Mean Age: 51.9 years<br><br>Median age 52 years<br>Male: 56.14%	14 Day Paper Food Record<br><br>Duration of Observation: 3 years	Children: Nil<br><br>Adolescents: Nil<br><br>Adults: 107/180	Baseline FR: 59.4%<br>Baseline FFQ: Nil<br><br>Point 2 FR: Nil<br>Point 2 FFQ: Nil

					Total Participants: 180	Point 3 FR:<br>Point 3 FFQ: Nil
Laursen et al., 2021 [53]	Denmark, 2021	Cardiac Rehabilitation	Mean Age: 60.5 years<br><br>Male: % Unable to Ascertain	198-Item Paper Food Frequency Questionnaire<br><br>Duration of Observation: 3 Years	Children: Nil<br><br>Adolescents: Nil<br><br>Adults:<br>B:217/233<br>4wks: 186/202<br>6mths: 157/173<br><br>Total Participants: 233	Baseline FR: Nil<br>Baseline FFQ: 93.1%<br><br>Point 2 FR: Nil<br>Point 2 FFQ: 92.1%<br><br>Point 3 FR: Nil<br>Point 3 FFQ: 91.0%
Lee et al., 2020 [54]	South Korea, 2020	Chronic Kidney Disease (CKD)	Mean Age: 59.9 ± 12.1 years.<br><br>Male: % Unable to Ascertain	3 Day Paper Food Record<br><br>Duration of Observation: 1 year	Children: Nil<br><br>Adolescents: Nil<br><br>Adults: 256/350<br><br>Total Participants: 350	Baseline FR: 73.1%<br>Baseline FFQ: Nil<br><br>Point 2 FR: Nil<br>Point 2 FFQ: Nil<br><br>Point 3 FR: Nil<br>Point 3 FFQ: Nil
Lei et al., 2022 [55]	China, 2022	Breast Cancer	Mean Age: Unable to Ascertain<br><br>Male: 0%	109- Item Paper Food Frequency Questionnaire. European Organisation for Research and Treatment of Cancer Quality of Life Questionnaire Core 30 (EORTC QLQ-C30)<br><br>Duration of Observation: 5 years	Children: Nil<br><br>Adolescents: Nil<br><br>Adults:<br>B18 mths: 1310/1462<br>36 mths: 1079/1462<br>5 years: 1095/1462<br><br>Total Participants: 1462	Baseline FR: Nil<br>Baseline FFQ:90.0%<br><br>Point 2 FR: Nil<br>Point 2 FFQ: 74.0%<br><br>Point 3 FR: Nil<br>Point 3 FFQ:75.0%
Leroux et al., 2015 [56]	Canada, 2015	Type 1 Diabetes Mellitus (T1DM)	Mean Age: 44.0 ± 12.5 Years<br><br>Male: 49%	3 Day Paper Food Record<br><br>Duration of Observation: 2 Years	Children: Nil<br><br>Adolescents: Nil<br><br>Adults: 118/124	Baseline FR: 95.2%<br>Baseline FFQ: Nil<br><br>Point 2 FR: Nil<br>Point 2 FFQ: Nil

					Total Participants: 124	Point 3 FR: Nil<br>Point 3 FFQ: Nil
Lin et al., 2019 [57]	Taiwan, 2019	Kidney Transplant	Mean Age: 49.7 ± 12.5 years<br><br>Male: 53.3%	3-Day Paper Food Record (2 Weekdays and 1 day on the Weekend)<br><br>Duration of Observation: 2 Years	Children: Nil<br><br>Adolescents: Nil<br><br>Adults: 90/90<br><br>Total Participants: 90	Baseline FR: 100%<br>Baseline FFQ: Nil<br><br>Point 2 FR: Nil<br>Point 2 FFQ: Nil<br><br>Point 3 FR: Nil<br>Point 3 FFQ: Nil
Li et al., 2022 [58]	China, 2022	Gout	Mean Age: 42.7 ± 14.2 years<br><br>Male: 94.6%	10-Item Paper Food Frequency Questionnaire<br><br>Duration of Observation: Unable to Ascertain	Children: Nil<br><br>Adolescents: Nil<br><br>Adults: 659/666<br><br>Total Participants: 666	Baseline FR: Nil<br>Baseline FFQ: 99.0%<br><br>Point 2 FR: Nil<br>Point 2 FFQ: Nil<br><br>Point 3 FR: Nil<br>Point 3 FFQ: Nil
Mardas et al., 2015 [59]	Poland, 2015	Ovarian Cancer	Mean Age: 57.9 ± 10.1 years<br><br>Male: 0 %	(1) 101-Item Paper Food Frequency Questionnaire<br><br>(2) 7-day Paper Food Record.<br><br>Duration of Observation: 7 days	Children: Nil<br><br>Adolescents: Nil<br><br>Adults:<br>(1) 44/61<br>(2) 44/61<br><br>Total Participants: 61	Baseline FR: 72.1%<br>Baseline FFQ: 72.1%<br><br>Point 2 FR: Nil<br>Point 2 FFQ: Nil<br><br>Point 3 FR: Nil<br>Point 3 FFQ: Nil
Mardas et al., 2016 [60]	Poland, 2016	Ovarian Cancer	Mean Age: 59.7 Years<br><br>Male: 0%	3-Day Paper Food Record<br><br>Duration of Observation: 6 months	Children: Nil<br><br>Adolescents: Nil<br><br>Adults: 41/55<br><br>Total Participants: 55	Baseline FR: 74.5%<br>Baseline FFQ: Nil<br><br>Point 2 FR: Nil<br>Point 2 FFQ: Nil<br><br>Point 3 FR: Nil<br>Point 3 FFQ: Nil

Mazzeo et al., 2016 [61]	Italy, 2016	Coeliac Disease	Mean Age: 41.6 ± 12.2 Years<br><br>Male: % Unclear	(1) 7 Day Paper Weighed Food Record<br><br>(2) 188-Item Paper Food Frequency Questionnaire (EPIC FFQ developed for North Central Italy- Coeliac Population)	Children: Nil<br><br>Adolescents: Nil<br>Adults:<br>(1) 107/200<br>(2) 134/200<br><br>Total Participants: 200	Baseline FR: 53.5%<br>Baseline FFQ:67.0%<br><br>Point 2 FR: Nil<br>Point 2 FFQ: Nil<br><br>Point 3 FR: Nil<br>Point 3 FFQ: Nil
Mehta et al., 2023 [62]	United States of America, 2023	Coeliac Disease	Mean Age: Unable to Ascertain<br><br>Male: 37.0%	3 Day Paper Food Record (2 Weekdays and 1 day on the Weekend)<br><br>Duration of Observation: 3 days	Children:<br>B: 400/477<br>P2: 296/400<br><br>Adolescents: Nil<br><br>Adults: Nil<br><br>Total Participants: 477	Baseline FR: 84.0%<br>Baseline FFQ: Nil<br><br>Point 2 FR: 74.0%<br>Point 2 FFQ: Nil<br><br>Point 3 FR: Nil<br>Point 3 FFQ: Nil
Milajerdi et al., 2022 [63]	Iran, 2022	Glioma	Mean Age: 43.43 ± 14.61 Years<br><br>Male: 58.6%	123-Item Food Frequency Questionnaire (Unclear Format)<br><br>Duration of Observation: 2 Years	Children: Nil<br><br>Adolescents: Nil<br><br>Adults: 123/128<br><br>Total Participants: 128	Baseline FR: Nil<br>Baseline FFQ:96.1%<br><br>Point 2 FR: Nil<br>Point 2 FFQ: Nil<br><br>Point 3 FR: Nil<br>Point 3 FFQ: Nil
Morton et al., 2020 [64]	New Zealand, 2020	Inflammatroy Bowel Disease (IBD)	Mean Age: 40.8 ± 14.9 Years<br><br>Male: 29%	142-Item Paper and Electronic Food Frequency Questionnaire<br><br>Duration of Observation: Unclear	Children: Nil<br><br>Adolescents: Nil<br><br>Adults: 233/254<br><br>Total Participants: 254	Baseline FR: Nil<br>Baseline FFQ: 92.0%<br><br>Point 2 FR: Nil<br>Point 2 FFQ: Nil<br><br>Point 3 FR: Nil<br>Point 3 FFQ: Nil
Na et al., 2021 [65]	South Korea, 2021	Irritable Bowel Syndrome (IBS)	Mean Age: 30.3 ± 7.3 Years	118- Item Electronic	Children: Nil	Baseline FR: Nil<br>Baseline FFQ:93.3%

			Male: 49.7%	Food Frequency Questionnaire<br><br>Duration of Observation: 5 months	Adolescents: Nil<br><br>Adults: 933/1000<br><br>Total Participants: 1000	Point 2 FR: Nil<br>Point 2 FFQ: Nil<br><br>Point 3 FR: Nil<br>Point 3 FFQ: Nil
Petrick et al., 2015 [66]	United States of America, 2015	Barrett's Oesophagus	Mean Age: Unable to Ascertain<br><br>Range: 20-80 years<br><br>Male: % Unable To Ascertain	131- Item Paper Food Frequency Questionnaire<br><br>Duration of Observation: 1 Year	Children: Nil<br><br>Adolescents: Nil<br><br>Adults: 177/193<br><br>Total Participants: 193	Baseline FR: Nil<br>Baseline FFQ:92.0%<br><br>Point 2 FR: Nil<br>Point 2 FFQ: Nil<br><br>Point 3 FR: Nil<br>Point 3 FFQ: Nil
Piotrowicz et al., 2015 [67]	Poland, 2015	Metabolic Syndrome	Mean Age: 48 ± 9 Years<br><br>Male:79.6%	24 Hour Paper Food Record<br><br>Duration of Observation: 24 Hours	Children: Nil<br><br>Adolescents: Nil<br><br>Adults: 105/113<br><br>Total Participants: 113	Baseline FR:93%<br>Baseline FFQ: Nil<br><br>Point 2 FR: Nil<br>Point 2 FFQ: Nil<br><br>Point 3 FR: Nil<br>Point 3 FFQ: Nil
Polderman et al., 2021 [68]	Canada, 2021	Nephrotic Syndrome	Mean Age: 4.8 years<br><br>Male: 56%	3 Day Paper Food Record (2 Weekdays and 1 day on the Weekend)<br><br>Duration of Observation: 3 days	Children: 53/76<br><br>Adolescents: Nil<br><br>Adults: Nil<br><br>Total Participants: 76	Baseline FR:70.0%<br>Baseline FFQ: Nil<br><br>Point 2 FR: Nil<br>Point 2 FFQ: Nil<br><br>Point 3 FR: Nil<br>Point 3 FFQ: Nil
Rej et al., 2021 [69]	United Kingdom, 2021	Irritable Bowel Syndrome (IBS)	Mean Age: 50 ± 16 years<br><br>Male: 25.4%	297-Item Food Frequency Questionnaire. (Unclear Format)<br><br>Duration of Observation: 3.7 years	Children: Nil<br><br>Adolescents: Nil<br><br>Adults: 346/606	Baseline FR: Nil<br>Baseline FFQ:57.1%<br><br>Point 2 FR: Nil<br>Point 2 FFQ: Nil

					Total Participants: 606	Point 3 FR: Nil<br>Point 3 FFQ: Nil
Shin et al., 2016 [70]	South Korea, 2016	Breast Cancer	Mean Age: Unable to Ascertain<br><br>Male: 0%	123-Item Paper Food Frequency Questionnaire.<br><br>Duration of Observation: 2 Years	Children: Nil<br><br>Adolescents: Nil<br><br>Adults: 192/219<br><br>Total Participants: 219	Baseline FR: Nil<br>Baseline FFQ: 88.0%<br><br>Point 2 FR: Nil<br>Point 2 FFQ: Nil<br><br>Point 3 FR: Nil<br>Point 3 FFQ: Nil
Shi et al., 2020 [71]	United States of America, 2020	Breast Cancer	Mean Age: 61 years<br><br>Male: 0%	139-Item Paper Food Frequency Questionnaire<br><br>Duration of Observation: 2 Years	Children: Nil<br><br>Adolescents: Nil<br><br>Adults:<br>B: 2865/4505<br>6 mths: 2733/4505<br>2 Years: 2067/4505<br><br>Total Participants: 4505	Baseline FR: Nil<br>Baseline FFQ:63.6%<br><br>Point 2 FR: Nil<br>Point 2 FFQ: 60.7%<br><br>Point 3 FR: Nil<br>Point 3 FFQ: 45.9%
Shu et al., 2017 [72]	Malaysia, 2017	Type 2 Diabetes Mellitus	Mean Age: 53.0 ± 9.4 Years<br><br>Male: 46.5%	122- Item Paper Food Frequency Questionnaire<br><br>Duration of Observation: Unclear	Children: Nil<br><br>Adolescents: Nil<br><br>Adults: 155/193<br><br>Total Participants: 193	Baseline FR:<br>Baseline FFQ:80.3%<br><br>Point 2 FR: Nil<br>Point 2 FFQ: Nil<br><br>Point 3 FR: Nil<br>Point 3 FFQ: Nil
Silveira et al., 2021 [73]	United States of America, 2021	Multiple Sclerosis (MS)	Mean Age: 61 ± 10 years<br><br>Male: 26%	263-Item Electronic Diet History Food Frequency Questionnaire (DHQ III)<br><br>Duration of Observation: Unclear	Children: Nil<br><br>Adolescents: Nil<br><br>Adults:<br>(1) 154/169<br>(2) 159/169	Baseline FR: Nil<br>Baseline FFQ:<br>(1) 91.1%<br>(2) 94.1%<br><br>Point 2 FR: Nil<br>Point 2 FFQ: Nil

					Total Participants: 169	Point 3 FR: Nil<br>Point 3 FFQ: Nil
Silviera et al., 2025 [74]	United States of America, 2025	Multiple Sclerosis (MS)	Mean Age: 39.8 ± 9.6 Years<br><br>Male: 14.3%	Electronic Automated Self-Administered 24 h (ASA24) Dietary Assessment Tool (version 2018)<br><br>Duration of Observation: 1 Year	Children: Nil<br><br>Adolescents: Nil<br><br>Adults: 28/30<br><br>Total Participants: 30	Baseline FR: 93.3%<br>Baseline FFQ: Nil<br><br>Point 2 FR: Nil<br>Point 2 FFQ: Nil<br><br>Point 3 FR: Nil<br>Point 3 FFQ: Nil
Smith et al., 2024 [75]	United Kingdom, 2024	Cancer: (Breast, Prostate and Colon)	Mean Age: 67.4 ± 11.8 Years<br><br>Male: 43.8%	Dietary Instrument for Nutrition Education Food Frequency Questionnaire (DINE FFQ)<br>(Unclear Format)<br><br>Duration of Observation: Unclear	Children: Nil<br><br>Adolescents: Nil<br><br>Adults: 5835/13,500<br><br>Total Participants: 13,500	Baseline FR: Nil<br>Baseline FFQ: 43.2%<br><br>Point 2 FR: Nil<br>Point 2 FFQ: Nil<br><br>Point 3 FR: Nil<br>Point 3 FFQ: Nil
Subih et al., 2023 [76]	Jordan, 2023	Chemotherapy	Mean Age: Unable to Ascertain<br><br>Male: 25.3%	3-Day Paper Food Record. (2 Weekdays and 1 day on the Weekend)<br><br>Duration of Observation: 3 Days	Children: Nil<br><br>Adolescents: Nil<br><br>Adults: 75/75<br><br>Total Participants: 75	Baseline FR: 100%<br>Baseline FFQ: Nil<br><br>Point 2 FR: Nil<br>Point 2 FFQ: Nil<br><br>Point 3 FR: Nil<br>Point 3 FFQ: Nil
Taha et al., 2022 [77]	United States of America, 2022	Head and Neck Cancer	Mean Age: 61.1 Years<br><br>Male: 75.3%	131-item paper self-administered Harvard Food Frequency Questionnaire<br><br>Duration of Observation: Unclear	Children: Nil<br><br>Adolescents: Nil<br><br>Adults: 582/1137<br><br>Total Participants: 1137	Baseline FR:<br>Baseline FFQ:51.2%<br><br>Point 2 FR: Nil<br>Point 2 FFQ: Nil<br><br>Point 3 FR: Nil<br>Point 3 FFQ: Nil

Tanaka et al., 2020 [78]	Japan, 2020	Type 2 Diabetes Mellitus	Mean Age: 60 years<br><br>Male: 55.0%	Self-reported Paper dietary history questionnaires<br><br>Duration of Observation: 3 Days	Children: Nil<br><br>Adolescents: Nil<br><br>Adults: 59/60<br><br>Total Participants: 60	Baseline FR: Nil<br>Baseline FFQ: 98.3%<br><br>Point 2 FR: Nil<br>Point 2 FFQ: Nil<br><br>Point 3 FR: Nil<br>Point 3 FFQ: Nil
Tasson et al., 2017 [79]	Italy, 2017	Inflammatory Bowel Disease (IBD)	Mean Age: 45.8 years<br><br>Male: 60.2%	146-Item Food Frequency Questionnaire.<br><br>Duration of Observation: 1 Year	Children: Nil<br><br>Adolescents: Nil<br><br>Adults: 103/130<br><br>Total Participants: 130	Baseline FR: Nil<br>Baseline FFQ: 79.2%<br><br>Point 2 FR: Nil<br>Point 2 FFQ: Nil<br><br>Point 3 FR: Nil<br>Point 3 FFQ: Nil
Teasdale et al., 2020 [80]	Australia, 2020	Severe Mental Illness	Mean Age: 19.7 ± 2.5 years<br><br>Male: 73.0%	120 Item Electronic Food Frequency Questionnaire.<br><br>Duration of Observation : Unclear	Children: Nil<br><br>Adolescents: Nil<br><br>Adults: 30/32<br><br>Total Participants: 32	Baseline FR: Nil<br>Baseline FFQ: 94.0%<br><br>Point 2 FR: Nil<br>Point 2 FFQ: Nil<br><br>Point 3 FR: Nil<br>Point 3 FFQ: Nil
Tedeschi et al., 2017 [81]	United States of America, 2017	Rheumatoid Arthritis	Mean Age: 65.0 Years<br><br>Male: 17%	20 Item-Paper Food Frequency Questionnaire.<br><br>Duration of Observation: Unclear	Children: Nil<br><br>Adolescents: Nil<br><br>Adults: 217/300<br><br>Total Participants: 300	Baseline FR:<br>Baseline FFQ: 72.3%<br><br>Point 2 FR: Nil<br>Point 2 FFQ: Nil<br><br>Point 3 FR: Nil<br>Point 3 FFQ: Nil
Thewjitcharoen et al., 2018 [82]	Thailand, 2018	Type 2 Diabetes Mellitus	Mean Age: 57.4 ± 10.9 years	3 Day Paper Food Record.(2 Weekdays and 1 day on the Weekend)	Children: Nil<br><br>Adolescents: Nil	Baseline FR: 70.1%<br>Baseline FFQ: Nil<br><br>Point 2 FR: Nil

			Male:47.4 %	Duration of Observation: 2 Years	Adults: 213/304<br><br>Total Participants: 304	Point 2 FFQ: Nil<br><br>Point 3 FR: Nil<br>Point 3 FFQ: Nil
Thomson et al., 2024 [83]	Australia, 2024	Type 1 Diabetes Mellitus	Mean Age: 31.7 ± 4.6 Years<br><br>Male: 0%	74-Item Electronic Food Frequency Questionnaire<br><br>Duration of Observation: 10 months	Children: Nil<br><br>Adolescents: Nil<br><br>Adults: 615/725<br><br>Total Participants: 725	Baseline FR: Nil<br>Baseline FFQ: 85.0%<br><br>Point 2 FR: Nil<br>Point 2 FFQ: Nil<br><br>Point 3 FR: Nil<br>Point 3 FFQ: Nil
Tseng et al., 2022 [84]	China, 2022	Human Immunodeficiency Virus (HIV)	Mean Age: Unclear<br><br>Median Age<br><br>Male: 89.0%	107 -Item Electronic Food Frequency Questionnaire<br><br>Duration of Observation: 3 months	Children: Nil<br><br>Adolescents: Nil<br><br>Adults: 83/127<br><br>Total Participants: 127	Baseline FR: Nil<br>Baseline FFQ:65.4%<br><br>Point 2 FR: Nil<br>Point 2 FFQ: Nil<br><br>Point 3 FR: Nil<br>Point 3 FFQ: Nil
Van Blarigan et al., 2020 [85]	United States of America, 2020	Colorectal Cancer	Mean Age: 59 years<br><br>Male:59.0 %	130-Item Food Frequency Questionnaire (Unclear Format)<br><br>Duration of Observation: 3 months	Children: Nil<br><br>Adolescents: Nil<br><br>Adults:<br>(1) 1354/2334<br>(2) 1284/2334<br><br>Total Participants: 2334	Baseline FR: Nil<br>Baseline FFQ:<br>(1) 58.0%<br>(2) 55.0%<br><br>Point 2 FR: Nil<br>Point 2 FFQ: Nil<br><br>Point 3 FR:<br>Point 3 FFQ:
Van Lanen et al., 2024 [86]	Netherlands, 2024	Colorectal Cancer	Mean Age: 66 Years<br><br>Male: 62.2 %	204-Item Food Frequency Questionnaire (Unclear Format)	Children: Nil<br><br>Adolescents: Nil<br><br>Adults: 2383/2544	Baseline FR: Nil<br>Baseline FFQ: 94.0%<br><br>Point 2 FR: Nil<br>Point 2 FFQ: Nil

				Duration of Observation: 1 month	Total Participants: 2544	Point 3 FR: Nil<br>Point 3 FFQ: Nil
Wu et al., 2021 [87]	Netherlands, 2021	Colorectal Cancer Survivors	Mean Age: 70 ± 9.0 Years<br><br>Male: 62.8%	7 Day Paper Food Record<br><br>Duration of Observation: Unclear	Children: Nil<br><br>Adolescents: Nil<br><br>Adults: 154/155<br><br>Total Participants: 155	Baseline FR: 99.4%<br>Baseline FFQ: Nil<br><br>Point 2 FR: Nil<br>Point 2 FFQ: Nil<br><br>Point 3 FR: Nil<br>Point 3 FFQ: Nil
Zeng et al., 2017 [88]	China, 2017	Kidney Stones	Mean Age: 48 years<br><br>Male:45.2 %	109- Item Paper Food Frequency Questionnaire<br><br>Duration of Observation: 1 Year	Children: Nil<br><br>Adolescents: Nil<br><br>Adults: 9322/9686<br><br>Total Participants: 9686	Baseline FR: Nil<br>Baseline FFQ: 96.2%<br><br>Point 2 FR: Nil<br>Point 2 FFQ: Nil<br><br>Point 3 FR: Nil<br>Point 3 FFQ: Nil

References:

1. Adanan, N. I. H., M. S. Md Ali, J. H. Lim, N. F. Zakaria, C. T. S. Lim, R. Yahya, A. H. Abdul Gafor, T. Karupaiah and Z. M. Daud. "Investigating physical and nutritional changes during prolonged intermittent fasting in hemodialysis patients: A prospective cohort study." *Journal of Renal Nutrition* 30 (2020): e15-e26. 10.1053/j.jrn.2019.06.003. https://www.scopus.com/inward/record.uri?eid=2-s2.0-85070522588&doi=10.1053%2fj.jrn.2019.06.003&partnerID=40&md5=4514229e68d6f6f501d1776edfcc6bfc.
2. Affret, A., S. Wagner, D. El Fatouhi, C. Dow, E. Correia, M. Niravong, F. Clavel-Chapelon, J. De Chefdebien, D. Fouque, B. Stengel, *et al.* "Validity and reproducibility of a short food frequency questionnaire among patients with chronic kidney disease." *BMC Nephrology* 18 (2017): 10.1186/s12882-017-0695-2. https://www.scopus.com/inward/record.uri?eid=2-s2.0-85029518222&doi=10.1186%2fs12882-017-0695-2&partnerID=40&md5=ff51223d279ac6ac4149d0e0be498265.
3. Ahola, A. J., C. Forsblom and P. H. Groop. "Adherence to special diets and its association with meeting the nutrient recommendations in individuals with type 1 diabetes." *Acta Diabetologica* 55 (2018): 843-51. 10.1007/s00592-018-1159-2. https://www.scopus.com/inward/record.uri?eid=2-s2.0-85047161885&doi=10.1007%2fs00592-018-1159-2&partnerID=40&md5=7d56545d0cf248b955e2925d9fb95594.

4. Ahola, A. J., C. M. Forsblom, V. Harjutsalo and P. H. Groop. "Nut consumption is associated with lower risk of metabolic syndrome and its components in type 1 diabetes." *Nutrients* 13 (2021): 10.3390/nu13113909. https://www.scopus.com/inward/record.uri?eid=2-s2.0-85118372100&doi=10.3390%2fnu13113909&partnerID=40&md5=4363363a131eeceab14de5543050fad3.
5. Amalia, R. I. and A. Davenport. "Estimated dietary sodium intake in peritoneal dialysis patients using food frequency questionnaires and total urinary and peritoneal sodium losses and assessment of extracellular volumes." *European Journal of Clinical Nutrition* 73 (2019): 105-11. 10.1038/s41430-018-0259-y. https://www.scopus.com/inward/record.uri?eid=2-s2.0-85050692907&doi=10.1038%2fs41430-018-0259-y&partnerID=40&md5=d91a5bbb7f0941ff43b7ab61ef05d41a.
6. Aponte, C. A. and R. G. Romanczyk. "Assessment of feeding problems in children with autism spectrum disorder." *Research in Autism Spectrum Disorders* 21 (2016): 61-72. 10.1016/j.rasd.2015.09.007. https://www.scopus.com/inward/record.uri?eid=2-s2.0-84945190001&doi=10.1016%2fj.rasd.2015.09.007&partnerID=40&md5=2f9dab46eec7debd205d882af025dd2c.
7. Arthur, A. E., A. M. Goss, W. Demark-Wahnefried, A. M. Mondul, K. R. Fontaine, Y. T. Chen, W. R. Carroll, S. A. Spencer, L. Q. Rogers, L. S. Rozek*, et al.* "Higher carbohydrate intake is associated with increased risk of all-cause and disease-specific mortality in head and neck cancer patients: Results from a prospective cohort study." *Int J Cancer* 143 (2018): 1105-13. 10.1002/ijc.31413.
8. Bail, J. R., S. V. Bail, J. Cagle, K. Tiesi, J. Caffey, M. Bakitas and W. Demark-Wahnefried. "Health behaviors and well-being among those "living" with metastatic cancer in alabama." *Supportive Care in Cancer* 30 (2022): 1689-701. 10.1007/s00520-021-06583-1. https://www.scopus.com/inward/record.uri?eid=2-s2.0-85115703930&doi=10.1007%2fs00520-021-06583-1&partnerID=40&md5=957457c5584055976cb6808889e9b92c.
9. Baleato, C. L., J. J. A. Ferguson, C. Oldmeadow, G. D. Mishra and M. L. Garg. "Plant-based dietary patterns versus meat consumption and prevalence of impaired glucose intolerance and diabetes mellitus: A cross-sectional study in australian women." *Nutrients* 14 (2022): 10.3390/nu14194152. https://www.scopus.com/inward/record.uri?eid=2-s2.0-85139858951&doi=10.3390%2fnu14194152&partnerID=40&md5=f412e6ab59bbb999458732225f162f2c.
10. Basu, A., A. C. Alman and J. K. Snell-Bergeon. "Dietary fiber intake and glycemic control: Coronary artery calcification in type 1 diabetes (cacti) study." *Nutrition Journal* 18 (2019): 10.1186/s12937-019-0449-z. https://www.scopus.com/inward/record.uri?eid=2-s2.0-85063960933&doi=10.1186%2fs12937-019-0449-z&partnerID=40&md5=1b3f35a1d8ad86e062d41118b6d499f4.
11. Basu, A., A. C. Alman and J. K. Snell-Bergeon. "Associations of dietary patterns and nutrients with glycated hemoglobin in participants with and without type 1 diabetes." *Nutrients* 13 (2021): 10.3390/nu13031035. https://www.scopus.com/inward/record.uri?eid=2-s2.0-85102814933&doi=10.3390%2fnu13031035&partnerID=40&md5=db7811b096c2a5173d86cbb0dab25fbb.
12. Beeren, I., L. de Goeij, R. Dandis, N. Vidra, M. van Zutphen, J. A. Witjes, E. Kampman, L. A. L. M. Kiemeney and A. Vrieling. "Limited changes in lifestyle behaviours after non-muscle invasive bladder cancer diagnosis." *Cancers* 14 (2022): 10.3390/cancers14040960. https://www.scopus.com/inward/record.uri?eid=2-s2.0-85124456153&doi=10.3390%2fcancers14040960&partnerID=40&md5=d54bb0c8ced060ada3bf6dab4b760a81.

13. Beiner, C., M. M. Qureshi, J. Zhao, B. Hu, R. Jimenez and A. E. Hirsch. "Depression and anxiety among english- and spanish-speaking patients with breast cancer receiving radiation therapy." *Int J Radiat Oncol Biol Phys* 119 (2024): 185-92. 10.1016/j.ijrobp.2023.11.049.
14. Belle, F. N., A. Chatelan, R. Kasteler, L. Mader, I. Guessous, M. Beck-Popovic, M. Ansari, C. E. Kuehni and M. Bochud. "Dietary intake and diet quality of adult survivors of childhood cancer and the general population: Results from the sccss-nutrition study." *Nutrients* 13 (2021): 10.3390/nu13061767. https://www.scopus.com/inward/record.uri?eid=2-s2.0-85106310993&doi=10.3390%2fnu13061767&partnerID=40&md5=42591e4934dcb02341ecc8189478d363.
15. Birketvedt, K., A. Mikkelsen, L. L. Klingen, C. Henriksen, I. B. Helland and R. Emblem. "Nutritional status in adolescents with esophageal atresia." *J Pediatr* 218 (2020): 130-37. 10.1016/j.jpeds.2019.11.034.
16. Black, L. J., S. Hetherton, M. Forkan, E. G. Gonzales, J. B. Smith, A. Daly, R. M. Lucas and A. Langer-Gould. "An exploratory study of diet in childhood and young adulthood and adult-onset multiple sclerosis." *Multiple Sclerosis Journal* 27 (2021): 1611-14. 10.1177/1352458520986964. https://www.scopus.com/inward/record.uri?eid=2-s2.0-85099705009&doi=10.1177%2f1352458520986964&partnerID=40&md5=41a10ab8a0cd646123a3a5d0aedd28b5.
17. Bolte, L. A., K. A. Lee, J. R. Björk, E. R. Leeming, M. J. E. Campmans-Kuijpers, J. J. De Haan, A. V. Vila, A. Maltez-Thomas, N. Segata, R. Board*, et al.* "Association of a mediterranean diet with outcomes for patients treated with immune checkpoint blockade for advanced melanoma." *JAMA Oncology* 9 (2023): 705-09. 10.1001/jamaoncol.2022.7753. https://www.scopus.com/inward/record.uri?eid=2-s2.0-85159765905&doi=10.1001%2fjamaoncol.2022.7753&partnerID=40&md5=f8a59f8e0349fdd2335b6c6d9928da7d.
18. Boucher, B. A., S. Wanigaratne, S. A. Harris and M. Cotterchio. "Postdiagnosis isoflavone and lignan intake in newly diagnosed breast cancer patients: Cross-sectional survey shows considerable intake from previously unassessed high-lignan foods." *Current Developments in Nutrition* 2 (2018): https://www.scopus.com/inward/record.uri?eid=2-s2.0-85063312014&partnerID=40&md5=82c2487e03c9dbe190ed2755b0e2807a.
19. Bredin, C., S. Naimimohasses, S. Norris, C. Wright, N. Hancock, K. Hart and J. B. Moore. "Development and relative validation of a short food frequency questionnaire for assessing dietary intakes of non-alcoholic fatty liver disease patients." *European Journal of Nutrition* 59 (2020): 571-80. 10.1007/s00394-019-01926-5. https://www.scopus.com/inward/record.uri?eid=2-s2.0-85062148721&doi=10.1007%2fs00394-019-01926-5&partnerID=40&md5=97d511cc7089fe8f821c8e118f9b2024.
20. Chhabra, R. and A. Davenport. "Is increased subjective thirst associated with greater interdialytic weight gains, extracellular fluid and dietary sodium intake?" *Artificial Organs* 48 (2024): 91-97. 10.1111/aor.14657. https://www.scopus.com/inward/record.uri?eid=2-s2.0-85175378892&doi=10.1111%2faor.14657&partnerID=40&md5=5dadde7bc97bd1e7aebac2d985428559.
21. Coe, S., S. L. Spruzen, C. Sanchez, H. Izadi and H. Dawes. "A cross-sectional feasibility study of nutrient intake patterns in people with parkinson's compared to government nutrition guidelines." *J Am Coll Nutr* 39 (2020): 187-91. 10.1080/07315724.2019.1633440.
22. Conley, M., K. L. Campbell, C. M. Hawley, N. M. Lioufas, G. J. Elder, S. V. Badve, E. Pedagogos, E. Milanzi, E. M. Pascoe, A. Valks*, et al.* "Relationship between dietary phosphate intake and biomarkers of bone and mineral metabolism in australian adults with chronic kidney disease." *J Ren Nutr* 32 (2022): 58-67. 10.1053/j.jrn.2021.07.004.

23. Cooke, Z. M., S. M. Resciniti, B. J. Wright, M. W. Hale, C. K. Yao, C. J. Tuck and J. R. Biesiekierski. "Association between dietary factors, symptoms, and psychological factors in adults with dyspepsia: A cross-sectional study." *Neurogastroenterology and Motility* 35 (2023): 10.1111/nmo.14684. https://www.scopus.com/inward/record.uri?eid=2-s2.0-85173553019&doi=10.1111%2fnmo.14684&partnerID=40&md5=ffe9ead908e24afe527e372dc05b1b79.
24. Crowder, S. L., Z. Li, K. P. Sarma and A. E. Arthur. "Chronic nutrition impact symptoms are associated with decreased functional status, quality of life, and diet quality in a pilot study of long-term post-radiation head and neck cancer survivors." *Nutrients* 13 (2021): 10.3390/nu13082886. https://www.scopus.com/inward/record.uri?eid=2-s2.0-85113806378&doi=10.3390%2fnu13082886&partnerID=40&md5=8fc2c54be99e383e8c4d27102ac9e279.
25. Dewinter, L., K. Casteels, K. Corthouts, K. Van De Kerckhove, K. Van Der Vaerent, K. Vanmeerbeeck and C. Matthys. "Dietary intake of non-nutritive sweeteners in type 1 diabetes mellitus children." *Food Additives and Contaminants - Part A Chemistry, Analysis, Control, Exposure and Risk Assessment* 33 (2015): 19-26. 10.1080/19440049.2015.1112039. https://www.scopus.com/inward/record.uri?eid=2-s2.0-84946935036&doi=10.1080%2f19440049.2015.1112039&partnerID=40&md5=d590a88a02968edb20aafc7d2c0e3e76.
26. Dinparast, F., A. Sharifi, S. Moradi, M. Alipour and B. Alipour. "The associations between dietary pattern of chronic obstructive pulmonary disease patients and depression: A cross-sectional study." *BMC Pulmonary Medicine* 21 (2021): 10.1186/s12890-020-01383-5. https://www.scopus.com/inward/record.uri?eid=2-s2.0-85098796061&doi=10.1186%2fs12890-020-01383-5&partnerID=40&md5=2ec7546e839acd2c117ecb3c7c2004aa.
27. Dolovich, C., L. A. Shafer, K. Vagianos, K. Witges, L. E. Targownik and C. N. Bernstein. "The complex relationship between diet, symptoms, and intestinal inflammation in persons with inflammatory bowel disease: The manitoba living with ibd study." *Journal of Parenteral and Enteral Nutrition* 46 (2022): 867-77. 10.1002/jpen.2257. https://www.scopus.com/inward/record.uri?eid=2-s2.0-85115306889&doi=10.1002%2fjpen.2257&partnerID=40&md5=bf1be726cd2fbb3eba49465515f6dab7.
28. Dratsky, D., E. McGillivray, J. Mittal, E. A. Handorf, G. Berardi, I. Astsaturov, M. J. Hall, M. C. Yeh, R. Jain and C. Y. Fang. "Food insecurity and dietary quality in african american patients with gastrointestinal cancers: An exploratory study." *Nutrients* 16 (2024): 10.3390/nu16183057.
29. Drzymała-Czyz, S., Ł. Kałuzny, P. Krzyzanowska-Jankowska, D. Walkowiak, R. Mozrzymas and J. Walkowiak. "Deficiency of long-chain polyunsaturated fatty acids in phenylketonuria: A cross-sectional study." *Acta Biochimica Polonica* 65 (2018): 303-08. 10.18388/abp.2018_2565. https://www.scopus.com/inward/record.uri?eid=2-s2.0-85048697024&doi=10.18388%2fabp.2018_2565&partnerID=40&md5=84f141ab0f8748c0fb496cb6bc1221b3.
30. Ericson, J., L. Lundell, M. Lindblad, F. Klevebro, M. Nilsson and I. Rouvelas. "Assessment of energy intake and total energy expenditure in a series of patients who have undergone oesophagectomy following neoadjuvant treatment." *Clinical Nutrition ESPEN* 37 (2020): 121-28. 10.1016/j.clnesp.2020.03.007. https://www.scopus.com/inward/record.uri?eid=2-s2.0-85082826754&doi=10.1016%2fj.clnesp.2020.03.007&partnerID=40&md5=18399c5b053a7d80109ffb8954346290.

31. Ewers, B., E. Trolle, S. S. Jacobsen, D. Vististen, T. P. Almdal, T. Vilsbøll and J. M. Bruun. "Dietary habits and adherence to dietary recommendations in patients with type 1 and type 2 diabetes compared with the general population in denmark." *Nutrition* 61 (2019): 49-55. 10.1016/j.nut.2018.10.021. https://www.scopus.com/inward/record.uri?eid=2-s2.0-85060336949&doi=10.1016%2fj.nut.2018.10.021&partnerID=40&md5=b4da7a7a81f1a3098ea0e83e15cc09ec.
32. Ferrari, A., A. M. de Carvalho, J. Steluti, J. Teixeira, D. M. L. Marchioni and S. Aguiar. "Folate and nutrients involved in the 1-carbon cycle in the pretreatment of patients for colorectal cancer." *Nutrients* 7 (2015): 4318-35. 10.3390/nu7064318. https://www.scopus.com/inward/record.uri?eid=2-s2.0-84931281789&doi=10.3390%2fnu7064318&partnerID=40&md5=0b654cf756d6243d90753a5424225b87.
33. Fisher, E. L., N. A. Weaver, A. L. Marlow, B. R. King and C. E. Smart. "Macronutrient intake in children and adolescents with type 1 diabetes and its association with glycemic outcomes." *Pediatric Diabetes* 2023 (2023): 10.1155/2023/7102890. https://www.scopus.com/inward/record.uri?eid=2-s2.0-85179099799&doi=10.1155%2f2023%2f7102890&partnerID=40&md5=a9e119ae304c59dd6c89beb0fdafc344.
34. Ganguzza, L., C. Ngai, L. Flink, K. Woolf, Y. Guo, E. Gianos, J. Burdowski, J. Slater, V. Acosta, T. Shephard*, et al.* "Association between diet quality and measures of body adiposity using the rate your plate survey in patients presenting for coronary angiography." *Clin Cardiol* 41 (2018): 126-30. 10.1002/clc.22843.
35. Gilbertson, H. R., K. Reed, S. Clark, K. L. Francis and F. J. Cameron. "An audit of the dietary intake of australian children with type 1 diabetes." *Nutrition and Diabetes* 8 (2018): 10.1038/s41387-018-0021-5. https://www.scopus.com/inward/record.uri?eid=2-s2.0-85044226999&doi=10.1038%2fs41387-018-0021-5&partnerID=40&md5=2feb30ee0287c7754d12f1039ade0409.
36. Gingras, V., C. Leroux, K. Desjardins, V. Savard, S. Lemieux, R. Rabasa-Lhoret and I. Strychar. "Association between cardiometabolic profile and dietary characteristics among adults with type 1 diabetes mellitus." *J Acad Nutr Diet* 115 (2015): 1965-74. 10.1016/j.jand.2015.04.012.
37. Godny, L., N. Maharshak, L. Reshef, I. Goren, L. Yahav, N. Fliss-Isakov, U. Gophna, H. Tulchinsky and I. Dotan. "Fruit consumption is associated with alterations in microbial composition and lower rates of pouchitis." *Journal of Crohn's and Colitis* 13 (2019): 1265-72. 10.1093/ecco-jcc/jjz053. https://www.scopus.com/inward/record.uri?eid=2-s2.0-85067186376&doi=10.1093%2fecco-jcc%2fjjz053&partnerID=40&md5=63780dd93af8936124693ffee18b42f9.
38. Gregg, J. R., J. Zheng, D. S. Lopez, C. Reichard, G. Browman, B. Chapin, J. Kim, J. Davis and C. R. Daniel. "Diet quality and gleason grade progression among localised prostate cancer patients on active surveillance." *British Journal of Cancer* 120 (2019): 466-71. 10.1038/s41416-019-0380-2. https://www.scopus.com/inward/record.uri?eid=2-s2.0-85060732952&doi=10.1038%2fs41416-019-0380-2&partnerID=40&md5=7ea60b73db5203737781c99c3bd057bf.
39. Grieco, L. P., T. M. Brasky, C. K. Spees and J. L. Krok-Schoen. "The associations between dietary supplement use, diet quality, and health-related quality of life among older female cancer survivors." *Nutrition and Cancer* 74 (2022): 2829-37. 10.1080/01635581.2022.2035779.

https://www.scopus.com/inward/record.uri?eid=2-s2.0-85124256193&doi=10.1080%2f01635581.2022.2035779&partnerID=40&md5=a55c8d97a3052ec0020c8c9089134f32.

40. Helm, M. M., A. Basu, L. A. Richardson, L. C. Chien, K. Izuora, A. C. Alman and J. K. Snell-Bergeon. "Longitudinal three-year associations of dietary fruit and vegetable intake with serum hs-c-reactive protein in adults with and without type 1 diabetes." *Nutrients* 16 (2024): 10.3390/nu16132058. https://www.scopus.com/inward/record.uri?eid=2-s2.0-85198347029&doi=10.3390%2fnu16132058&partnerID=40&md5=e902dcae5e5b8b5fae0adaaa37617c64.

41. Horikawa, C., K. Tsuda, Y. Oshida, J. Satoh, Y. Hayashino, N. Tajima, R. Nishimura, H. Sone, D. Koya, K. Shikata*, et al.* "Dietary intake and physical activity in japanese patients with type 2 diabetes: The japan diabetes complication and its prevention prospective study (jdcp study 8)." *Diabetology International* 13 (2022): 344-57. 10.1007/s13340-022-00575-0. https://www.scopus.com/inward/record.uri?eid=2-s2.0-85132614435&doi=10.1007%2fs13340-022-00575-0&partnerID=40&md5=183491af63a7473b7e80ea3ecf144cb1.

42. Huisman, M. H. B., M. Seelen, P. T. C. Van Doormaal, S. W. De Jong, J. H. M. De Vries, A. J. Van Der Kooi, M. De Visser, H. J. Schelhaas, L. H. Van Den Berg and J. H. Veldink. "Effect of presymptomatic body mass index and consumption of fat and alcohol on amyotrophic lateral sclerosis." *JAMA Neurology* 72 (2015): 1155-62. 10.1001/jamaneurol.2015.1584. https://www.scopus.com/inward/record.uri?eid=2-s2.0-84944042440&doi=10.1001%2fjamaneurol.2015.1584&partnerID=40&md5=0e1bf3b886bff466fcfff19550267260.

43. Hu, J., C. La Vecchia, E. Negri, M. de Groh, H. Morrison and L. Mery. "Macronutrient intake and stomach cancer." *Cancer Causes and Control* 26 (2015): 839-47. 10.1007/s10552-015-0557-9. https://www.scopus.com/inward/record.uri?eid=2-s2.0-84929711905&doi=10.1007%2fs10552-015-0557-9&partnerID=40&md5=93a8df4e9d8f6adf6ff6f12b7972d75f.

44. Hussain, S. K., T. S. Dong, V. Agopian, J. R. Pisegna, F. A. Durazo, P. Enayati, V. Sundaram, J. N. Benhammou, M. Noureddin, G. Choi*, et al.* "Dietary protein, fiber and coffee are associated with small intestine microbiome composition and diversity in patients with liver cirrhosis." *Nutrients* 12 (2020): 10.3390/nu12051395. https://www.scopus.com/inward/record.uri?eid=2-s2.0-85084786589&doi=10.3390%2fnu12051395&partnerID=40&md5=b65cd21c620789951238be3e7a268f8a.

45. Ijpma, I., R. J. Renken, J. A. Gietema, R. H. J. A. Slart, M. G. J. Mensink, J. D. Lefrandt, G. J. Ter Horst and A. K. L. Reyners. "Changes in taste and smell function, dietary intake, food preference, and body composition in testicular cancer patients treated with cisplatin-based chemotherapy." *Clinical Nutrition* 36 (2017): 1642-48. 10.1016/j.clnu.2016.10.013. https://www.scopus.com/inward/record.uri?eid=2-s2.0-85006265392&doi=10.1016%2fj.clnu.2016.10.013&partnerID=40&md5=6d5538dc481a9154a2641d6d488a8cd9.

46. Ilari, S., L. Vitiello, P. Russo, S. Proietti, M. Milić, C. Muscoli, V. Cardaci, C. Tomino, G. Bonassi and S. Bonassi. "Daily vegetables intake and response to copd rehabilitation. The role of oxidative stress, inflammation and dna damage." *Nutrients* 13 (2021): 10.3390/nu13082787. https://www.scopus.com/inward/record.uri?eid=2-s2.0-85112348619&doi=10.3390%2fnu13082787&partnerID=40&md5=aacb937bde799f5d13fe27f5464de563.

47. Khatun, T., A. Hoque, K. S. Anwar, M. R. Sarker, F. Ara and D. Maqbool. "Dietary habits of patients with coronary artery disease in a tertiary-care hospital of bangladesh: A case-controlled study." *Journal of Health, Population and Nutrition* 40 (2021): 10.1186/s41043-

021-00226-1. https://www.scopus.com/inward/record.uri?eid=2-s2.0-85101838019&doi=10.1186%2fs41043-021-00226-1&partnerID=40&md5=1f2fc387db784e76cf1a68d8c53b6296.

48. Kiew, S. J., N. A. Mohd Taib, T. Islam and H. Abdul Majid. "Changes in dietary intake of breast cancer survivors: Early findings of a malaysian breast cancer prospective cohort study." *Nutrition and Cancer* 74 (2022): 2470-78. 10.1080/01635581.2021.2013508. https://www.scopus.com/inward/record.uri?eid=2-s2.0-85121450000&doi=10.1080%2f01635581.2021.2013508&partnerID=40&md5=cbfc0b10efd7f748f1f7ef6c26219bbd.

49. Klimek, A., C. Baerwald, M. Schwarz, F. Rutsch, K. G. Parhofer, U. Plöckinger, M. Heddrich-Ellerbrok, S. Vom Dahl, K. Schöne, M. Ott*, et al.* "Everyday life, dietary practices, and health conditions of adult pku patients: A multicenter, cross-sectional study." *Ann Nutr Metab* 76 (2020): 251-58. 10.1159/000510260.

50. Knoerl, R., R. Ploutz-Snyder, L. Smener, C. Tofthagen and S. Zick. "Association of chemotherapy-induced peripheral neuropathy with diet quality among post-treatment cancer survivors." *Nutrition and Cancer* 76 (2024): 717-25. 10.1080/01635581.2024.2364389. https://www.scopus.com/inward/record.uri?eid=2-s2.0-85197216201&doi=10.1080%2f01635581.2024.2364389&partnerID=40&md5=a388c3a507287e4bed12984d78e55d36.

51. Kristensen, M. B., C. L. Egholm, H. S. Vistisen, B. Borregaard, S. M. Bruvik, B. M. Bertelsen, E. Myrup, T. Mortensen, L. Viggers, R. E. Mols*, et al.* "Challenges and benefits of using the heartdiet food frequency questionnaire in cardiac rehabilitation practice." *Nutrition, Metabolism and Cardiovascular Diseases* 34 (2024): 1968-75. 10.1016/j.numecd.2024.04.016. https://www.scopus.com/inward/record.uri?eid=2-s2.0-85195645098&doi=10.1016%2fj.numecd.2024.04.016&partnerID=40&md5=61e1ad494167bad66cc60f640c2a96c7.

52. Lang, S., A. Martin, X. Zhang, F. Farowski, H. Wisplinghoff, M. J.G.T. Vehreschild, M. Krawczyk, A. Nowag, A. Kretzschmar, C. Scholz*, et al.* "Combined analysis of gut microbiota, diet and pnpla3 polymorphism in biopsy-proven non-alcoholic fatty liver disease." *Liver International* 41 (2021): 1576-91. 10.1111/liv.14899. https://www.scopus.com/inward/record.uri?eid=2-s2.0-85105243459&doi=10.1111%2fliv.14899&partnerID=40&md5=e4365f4ce77783c40aa4d91b1174958f.

53. Laursen, U. B., M. N. Johansen, A. M. Joensen, K. Overvad and M. L. Larsen. "Is cardiac rehabilitation equally effective in improving dietary intake in all patients with ischemic heart disease?" *Journal of the American College of Nutrition* 40 (2021): 33-40. 10.1080/07315724.2020.1755910. https://www.scopus.com/inward/record.uri?eid=2-s2.0-85086368945&doi=10.1080%2f07315724.2020.1755910&partnerID=40&md5=8d0454d0b17ee9352939462c876e5d44.

54. Lee, H., H. Kim, T. Y. Kim, H. Ryu, D. L. Ju, M. Jang, K. H. Oh, C. Ahn and S. N. Han. "Dietary assessment of korean non-dialysis chronic kidney disease patients with or without diabetes." *J Korean Med Sci* 35 (2020): e181. 10.3346/jkms.2020.35.e181.

55. Lei, Y. Y., S. C. Ho, C. Kwok, A. Cheng, K. L. Cheung, R. Lee, F. K. F. Mo and W. Yeo. "Association of high adherence to vegetables and fruits dietary pattern with quality of life among chinese women with early-stage breast cancer." *Quality of Life Research* 31 (2022): 1371-84. 10.1007/s11136-021-02985-0. https://www.scopus.com/inward/record.uri?eid=2-s2.0-85115099134&doi=10.1007%2fs11136-021-02985-0&partnerID=40&md5=5a85e9f858bda753bb2e224a90c9bbb2.

56. Leroux, C., V. Gingras, K. Desjardins, A. S. Brazeau, S. Ott-Braschi, I. Strychar and R. Rabasa-Lhoret. "In adult patients with type 1 diabetes healthy lifestyle associates with a better cardiometabolic profile." *Nutr Metab Cardiovasc Dis* 25 (2015): 444-51. 10.1016/j.numecd.2015.01.004.
57. Lin, I. H., T. C. Wong, S. W. Nien, Y. T. Chou, Y. J. Chiang, H. H. Wang and S. H. Yang. "Dietary compliance among renal transplant recipients: A single-center study in taiwan." *Transplant Proc* 51 (2019): 1325-30. 10.1016/j.transproceed.2019.02.026.
58. Li, Q. H., Y. W. Zou, S. Y. Lian, J. J. Liang, Y. F. Bi, C. Deng, Y. Q. Mo, K. M. Yang and L. Dai. "Sugar-sweeten beverage consumption is associated with more obesity and higher serum uric acid in chinese male gout patients with early onset." *Frontiers in Nutrition* 9 (2022): 10.3389/fnut.2022.916811. https://www.scopus.com/inward/record.uri?eid=2-s2.0-85134926611&doi=10.3389%2ffnut.2022.916811&partnerID=40&md5=3ac19c665c85bdc50097f236595664e3.
59. Mardas, M., M. Jamka, R. Mądry, J. Walkowiak, M. Krótkopad and M. Stelmach-Mardas. "Dietary habits changes and quality of life in patients undergoing chemotherapy for epithelial ovarian cancer." *Supportive Care in Cancer* 23 (2015): 1015-23. 10.1007/s00520-014-2462-2. https://www.scopus.com/inward/record.uri?eid=2-s2.0-84930509570&doi=10.1007%2fs00520-014-2462-2&partnerID=40&md5=f6b15d39b30a1269ca45f7f7879b1667.
60. Mardas, M., R. Mądry and M. Stelmach-Mardas. "Dietary intake variability in the cycle of cytotoxic chemotherapy." *Supportive Care in Cancer* 24 (2016): 2619-25. 10.1007/s00520-015-3072-3. https://www.scopus.com/inward/record.uri?eid=2-s2.0-84953258159&doi=10.1007%2fs00520-015-3072-3&partnerID=40&md5=6572c550a279f324687da8cf2bc30150.
61. Mazzeo, T., L. Roncoroni, V. Lombardo, C. Tomba, L. Elli, S. Sieri, S. Grioni, M. T. Bardella, C. Agostoni, L. Doneda*, et al.* "Evaluation of a modified italian european prospective investigation into cancer and nutrition food frequency questionnaire for individuals with celiac disease." *Journal of the Academy of Nutrition and Dietetics* 116 (2016): 1810-16. 10.1016/j.jand.2016.04.013. https://www.scopus.com/inward/record.uri?eid=2-s2.0-84969983892&doi=10.1016%2fj.jand.2016.04.013&partnerID=40&md5=611bcc3de7bc870c28f6b40c11531bdd.
62. Mehta, P., Q. Li, M. Stahl, U. Uusitalo, K. Lindfors, M. D. Butterworth, K. Kurppa, S. Virtanen, S. Koletzko, C. Aronsson*, et al.* "Gluten-free diet adherence in children with screening-detected celiac disease using a prospective birth cohort study." *PLoS ONE* 18 (2023): 10.1371/journal.pone.0275123. https://www.scopus.com/inward/record.uri?eid=2-s2.0-85147318953&doi=10.1371%2fjournal.pone.0275123&partnerID=40&md5=b3881fcef6496461ff5f7a2eefe97ecf.
63. Milajerdi, A., M. Shayanfar, S. Benisi-Kohansal, M. Mohammad-Shirazi, G. Sharifi, H. Tabibi and A. Esmaillzadeh. "A case-control study on dietary acid load in relation to glioma." *Nutrition and Cancer* 74 (2022): 1644-51. 10.1080/01635581.2021.1957134. https://www.scopus.com/inward/record.uri?eid=2-s2.0-85111690191&doi=10.1080%2f01635581.2021.1957134&partnerID=40&md5=f062133501000dd1fb80dd80e07cbb54.
64. Morton, H., K. C. Pedley, R. J. C. Stewart and J. Coad. "Inflammatory bowel disease: Are symptoms and diet linked?" *Nutrients* 12 (2020): 1-14. 10.3390/nu12102975. https://www.scopus.com/inward/record.uri?eid=2-s2.0-85091788868&doi=10.3390%2fnu12102975&partnerID=40&md5=37771c730cd500c9ff11fef59438a672.

65. Na, W., Y. Lee, H. Kim, Y. S. Kim and C. Sohn. "High-fat foods and fodmaps containing gluten foods primarily contribute to symptoms of irritable bowel syndrome in korean adults." *Nutrients* 13 (2021): 10.3390/nu13041308. https://www.scopus.com/inward/record.uri?eid=2-s2.0-85104110431&doi=10.3390%2fnu13041308&partnerID=40&md5=09f272b8eb78fecf12ff47309af4440d.
66. Petrick, J. L., S. E. Steck, P. T. Bradshaw, W. H. Chow, L. S. Engel, K. He, H. A. Risch, T. L. Vaughan and M. D. Gammon. "Dietary flavonoid intake and barrett's esophagus in western washington state." *Annals of Epidemiology* 25 (2015): 730-35.e2. 10.1016/j.annepidem.2015.05.010. https://www.scopus.com/inward/record.uri?eid=2-s2.0-84941189532&doi=10.1016%2fj.annepidem.2015.05.010&partnerID=40&md5=3c7e82f30b532b680f66c4a7d0060878.
67. Piotrowicz, K., E. Pałkowska, E. Bartnikowska, P. Krzesiński, A. Stańczyk, P. Biecek, A. Skrobowski and G. Gielerak. "Self-reported health-related behaviors and dietary habits in patients with metabolic syndrome." *Cardiology Journal* 22 (2015): 413-20. 10.5603/CJ.a2015.0020. https://www.scopus.com/inward/record.uri?eid=2-s2.0-84940530640&doi=10.5603%2fCJ.a2015.0020&partnerID=40&md5=8f5729990d9dafea184ffda66710c60a.
68. Polderman, N., M. Cushing, K. McFadyen, M. Catapang, R. Humphreys, C. Mammen, D. G. Matsell and T. Pediatric Nephrology Clinical Pathway Development. "Dietary intakes of children with nephrotic syndrome." *Pediatr Nephrol* 36 (2021): 2819-26. 10.1007/s00467-021-05055-2. https://www.ncbi.nlm.nih.gov/pubmed/33783623.
69. Rej, A., C. C. Shaw, R. L. Buckle, N. Trott, A. Agrawal, K. Mosey, K. Sanders, R. Allen, S. Martin, A. Newton*, et al.* "The low fodmap diet for ibs; a multicentre uk study assessing long term follow up." *Digestive and Liver Disease* 53 (2021): 1404-11. 10.1016/j.dld.2021.05.004. https://www.scopus.com/inward/record.uri?eid=2-s2.0-85107117027&doi=10.1016%2fj.dld.2021.05.004&partnerID=40&md5=43119ae2b1e871bdb688474ff42820f0.
70. Shin, W. K., S. Song, E. Hwang, H. G. Moon, D. Y. Noh and J. E. Lee. "Development of a ffq for breast cancer survivors in korea." *British Journal of Nutrition* 116 (2016): 1781-86. 10.1017/S000711451600372X. https://www.scopus.com/inward/record.uri?eid=2-s2.0-84995460834&doi=10.1017%2fS000711451600372X&partnerID=40&md5=25310da1fce3ff8b30aabd3a50d77ae0.
71. Shi, Z., A. Rundle, J. M. Genkinger, Y. K. Cheung, I. J. Ergas, J. M. Roh, L. H. Kushi, M. L. Kwan and H. Greenlee. "Distinct trajectories of fruits and vegetables, dietary fat, and alcohol intake following a breast cancer diagnosis: The pathways study." *Breast Cancer Research and Treatment* 179 (2020): 229-40. 10.1007/s10549-019-05457-9. https://www.scopus.com/inward/record.uri?eid=2-s2.0-85073953356&doi=10.1007%2fs10549-019-05457-9&partnerID=40&md5=f814f25639c8cca4e5abbea19590aeb3.
72. Shu, P. S., Y. M. Chan and S. L. Huang. "Higher body mass index and lower intake of dairy products predict poor glycaemic control among type 2 diabetes patients in malaysia." *PLoS ONE* 12 (2017): 10.1371/journal.pone.0172231. https://www.scopus.com/inward/record.uri?eid=2-s2.0-85014045164&doi=10.1371%2fjournal.pone.0172231&partnerID=40&md5=a254367c67d50b4d34065235377d7d95.

73. Silveira, S. L., B. Jeng, G. Cutter and R. W. Motl. "Diet quality assessment in wheelchair users with multiple sclerosis." *Nutrients* 13 (2021): 10.3390/nu13124352. https://www.scopus.com/inward/record.uri?eid=2-s2.0-85120558037&doi=10.3390%2fnu13124352&partnerID=40&md5=40dadc1e8342ea52b3a85cf4fbeff4ce.
74. Silveira, S. L., B. Jeng, B. A. Gower, G. R. Cutter and R. W. Motl. "Correlates of inaccuracy in reporting of energy intake among persons with multiple sclerosis." *Nutrients* 17 (2025): 10.3390/nu17030438. https://www.scopus.com/inward/record.uri?eid=2-s2.0-85217786909&doi=10.3390%2fnu17030438&partnerID=40&md5=3f375f04a2737dd71c6225dc0a253447.
75. Smith, S., A. Fisher, P. J. Lally, H. A. Croker, A. Roberts, R. E. Conway and R. J. Beeken. "Perceiving a need for dietary change in adults living with and beyond cancer: A cross-sectional study." *Cancer Medicine* 13 (2024): 10.1002/cam4.7073. https://www.scopus.com/inward/record.uri?eid=2-s2.0-85187442479&doi=10.1002%2fcam4.7073&partnerID=40&md5=27503c37e32394c98f45fe00479bff0c.
76. Subih, H. S., E. A. Al-Shwaiyat, N. Al-Bayyari, B. S. Obeidat, F. Abu-Farsakh and H. Bawadi. "Dietary intake is not associated with body composition nor with biochemical tests but with psychological status of cancer patients receiving chemotherapy." *Nutrients* 15 (2023): 10.3390/nu15245087. https://www.scopus.com/inward/record.uri?eid=2-s2.0-85180688688&doi=10.3390%2fnu15245087&partnerID=40&md5=5ace61f2404c6cc347f480a848d646d4.
77. Taha, H. M., L. S. Rozek, X. Chen, Z. Li, K. R. Zarins, A. N. Slade, G. T. Wolf and A. E. Arthur. "Risk of disease recurrence and mortality varies by type of fat consumed before cancer treatment in a longitudinal cohort of head and neck squamous cell carcinoma patients." *Journal of Nutrition* 152 (2022): 1298-305. 10.1093/jn/nxac032. https://www.scopus.com/inward/record.uri?eid=2-s2.0-85129997700&doi=10.1093%2fjn%2fnxac032&partnerID=40&md5=e6bdcfd926f64fee501f1733dd3f123a.
78. Tanaka, Y., T. Nakagami, J. Oya, C. Ukita-Shibasaki, Y. Takehana, S. Sasaki and T. Babazono. "Impact of body mass index and age on the relative accuracy of self-reported energy intakes among japanese patients with type 2 diabetes." *Diabetology International* 11 (2020): 360-67. 10.1007/s13340-020-00430-0. https://www.scopus.com/inward/record.uri?eid=2-s2.0-85082808368&doi=10.1007%2fs13340-020-00430-0&partnerID=40&md5=d5e4bf77520487446ee125c13091d09d.
79. Tasson, L., C. Canova, M. G. Vettorato, E. Savarino and R. Zanotti. "Influence of diet on the course of inflammatory bowel disease." *Digestive Diseases and Sciences* 62 (2017): 2087-94. 10.1007/s10620-017-4620-0. https://www.scopus.com/inward/record.uri?eid=2-s2.0-85019710574&doi=10.1007%2fs10620-017-4620-0&partnerID=40&md5=441027a630a2d541c41991b0251dc5dd.
80. Teasdale, S. B., T. L. Burrows, T. Hayes, C. Y. Hsia, A. Watkins, J. Curtis and P. B. Ward. "Dietary intake, food addiction and nutrition knowledge in young people with mental illness." *Nutrition and Dietetics* 77 (2020): 315-22. 10.1111/1747-0080.12550. https://www.scopus.com/inward/record.uri?eid=2-s2.0-85068134009&doi=10.1111%2f1747-0080.12550&partnerID=40&md5=35e4b1557b2cd52b40fcf0dbf110ded8.
81. Tedeschi, S. K., M. Frits, J. Cui, Z. Z. Zhang, T. Mahmoud, C. Iannaccone, T. C. Lin, K. Yoshida, M. E. Weinblatt, N. A. Shadick, *et al.* "Diet and rheumatoid arthritis symptoms: Survey results from a rheumatoid arthritis registry." *Arthritis Care and Research* 69 (2017):

1920-25. 10.1002/acr.23225. https://www.scopus.com/inward/record.uri?eid=2-s2.0-85035070530&doi=10.1002%2facr.23225&partnerID=40&md5=f38c8139ed3c21c1310b0454b8256f02.

82. Thewjitcharoen, Y., P. Chotwanvirat, A. Jantawan, N. Siwasaranond, S. Saetung, H. Nimitphong, T. Himathongkam and S. Reutrakul. "Evaluation of dietary intakes and nutritional knowledge in thai patients with type 2 diabetes mellitus." *Journal of Diabetes Research* 2018 (2018): 10.1155/2018/9152910. https://www.scopus.com/inward/record.uri?eid=2-s2.0-85060372932&doi=10.1155%2f2018%2f9152910&partnerID=40&md5=6040f1b2be3bd59c4c35f9a36e223224.

83. Thomson, R. L., J. D. Brown, H. Oakey, K. Palmer, P. Ashwood, M. A. S. Penno, K. J. McGorm, R. Battersby, P. G. Colman, M. E. Craig*, et al.* "Dietary patterns during pregnancy and maternal and birth outcomes in women with type 1 diabetes: The environmental determinants of islet autoimmunity (endia) study." *Diabetologia* 67 (2024): 2420-32. 10.1007/s00125-024-06259-5. https://www.scopus.com/inward/record.uri?eid=2-s2.0-85202966833&doi=10.1007%2fs00125-024-06259-5&partnerID=40&md5=8f195ac069211d81cd90eb72bc387c62.

84. Tseng, L. Y., W. Xie, W. Pan, H. Lyu, Z. Yu, W. Shi, Y. He, W. Chen, T. Li and E. Hsieh. "Validation of a six-item dietary calcium screening tool among hiv patients in china." *Public Health Nutrition* 24 (2021): 4786-95. 10.1017/S1368980021001427. https://www.scopus.com/inward/record.uri?eid=2-s2.0-85103757363&doi=10.1017%2fS1368980021001427&partnerID=40&md5=46191739f279eb30b5fd4d8eeba3cb33.

85. Van Blarigan, E. L., S. Zhang, F. S. Ou, A. Venlo, K. Ng, C. Atreya, K. Van Loon, D. Niedzwiecki, E. Giovannucci, E. G. Wolfe*, et al.* "Association of diet quality with survival among people with metastatic colorectal cancer in the cancer and leukemia b and southwest oncology group 80405 trial." *JAMA Network Open* 3 (2020): 10.1001/jamanetworkopen.2020.23500. https://www.scopus.com/inward/record.uri?eid=2-s2.0-85094935521&doi=10.1001%2fjamanetworkopen.2020.23500&partnerID=40&md5=3e74fa48a6f437d490fd4e8726daac16.

86. van Lanen, A. S., D. E. Kok, E. Wesselink, J. W. G. Derksen, A. M. May, K. C. Smit, M. Koopman, J. de Wilt, E. Kampman, F. J. B. van Duijnhoven*, et al.* "Associations between low- and high-fat dairy intake and recurrence risk in people with stage i–iii colorectal cancer differ by sex and primary tumour location." *International Journal of Cancer* 155 (2024): 828-38. 10.1002/ijc.34959. https://www.scopus.com/inward/record.uri?eid=2-s2.0-85192181875&doi=10.1002%2fijc.34959&partnerID=40&md5=4dbc9ce376113b72f1a050b8af7f596a.

87. Wu, W., M. J. L. Bours, A. Koole, M. F. Kenkhuis, S. J. P. M. Eussen, S. O. Breukink, F. J. van Schooten, M. P. Weijenberg and G. J. Hageman. "Cross-sectional associations between dietary daily nicotina-mide intake and patient-reported outcomes in colorectal cancer survivors, 2 to 10 years post-diagnosis." *Nutrients* 13 (2021): 10.3390/nu13113707. https://www.scopus.com/inward/record.uri?eid=2-s2.0-85117310592&doi=10.3390%2fnu13113707&partnerID=40&md5=f97a6c4ff19015540918cad394800e22.

88. Zeng, G., Z. Mai, S. Xia, Z. Wang, K. Zhang, L. Wang, Y. Long, J. Ma, Y. Li, S. P. Wan*, et al.* "Prevalence of kidney stones in china: An ultrasonography based cross-sectional study." *BJU Int* 120 (2017): 109-16. 10.1111/bju.13828.
