# Supplementary material for: Completion Rates of Food Frequency Questionnaires and Food Records in People with Chronic Conditions: Systematic Review and Meta-Analysis
Source: Nutrients. 2026 Jun 13;18(12):1922. doi: 10.3390/nu18121922 (PMC13306072; doi:10.3390/nu18121922)
Supplement: Supplementary file 1 [file nutrients-18-01922-s001.zip › supp Table S4 ROB ax.pdf]

Supplementary Table 4: Risk of Bias Assessment using Hoy assessment tool [1].

Study	External validity				Internal validity						Overall ROB
	1	2	3	4	5	6	7	8	9	10	Score /10
Adanan et al., 2020 [2]											Moderate risk 6
Affret et al., 2017 [3]											Low risk 8
Ahola et al., 2018 [4]											Low risk 8
Ahola et al., 2021 [5]											Moderate risk 6
Amalia et al., 2019 [6]											Low risk 8
Aponte et al., 2016 [7]											Low risk 9
Arthur et., 2018 [8]											Low risk 8
Bail et al., 2022 [9]											Low risk 9
Baleato et al., 2022 [10]											Low risk 10
Basu et al., 2019 [11]											Moderate risk 7
Basu et al., 2021 [12]											Moderate risk 7
Beeren et al., 2021 [13]											Low risk 9
Beiner et al., 2023 [14]											Moderate risk 6
Belle et al., 2021 [15]											Low risk 10
Birketvedt et al., 2020 [16]											Moderate risk 6
Black et al., 2021 [17]											Low risk 8
Bolte et al., 2023 [18]											Moderate risk 6
Boucher et al., 2018 [19]											Low risk 9
Bredin et al., 2020 [20]											Moderate risk 7
Chhabra et al., 2024 [21]											Moderate risk 6
Coe et al., 2020 [22]											Low risk 8
Conley et al., 2022 [23]											Moderate risk 6
Cooke et al., 2023 [24]											Moderate risk 6
Crowder et al., 2021 [25]											Moderate risk 6
Dewinter et al., 2015 [26]											Low risk 9
Dinparast et al., 2021 [27]											Low risk 8
Dolovich et al., 2022 [28]											Low risk 9
Dratsky et al., 2024 [29]											Moderate risk 6
Drzymała-Czyz et al., 2018 [30]											Moderate risk 6
Ericson et al., 2020 [31]											Moderate risk 6
Ewers et al., 2019 [32]											Low risk 10
Ferrari et al., 2015 [33]											Moderate risk 6
Fisher et al., 2023 [34]											Low risk 8
Ganguzza et al., 2018 [35]											Moderate risk 6
Gilbertson et al., 2018 [36]											Low risk 8
Gingras et al., 2015 [37]											Moderate risk 6
Godny et al., 2019 [38]											Moderate risk 6
Gregg et al., 2019 [39]											Moderate risk 7
Grieco et al., 2022 [40]											Moderate risk 6
Helm et al., 2024. [41]											Moderate risk 7
Horikawa et al., 2022 [42]											Low risk 8
Huisman et al., 2015											Low risk 10
Hu et al., 2015 [43]											Low risk 9
Hussain et al., 2020 [44]											Moderate risk 6

Ijpma et al., 2017 [45]											Moderate risk 6
Ilari et al., 2021 [46]											Moderate risk 7
Khatun et al., 2021 [47]											Moderate risk 7
Kiew et al., 2022 [48]											Moderate risk 8
Klimek et al., 2020 [49]											Low risk 9
Knoerl et al., 2024 [50]											Moderate risk 6
Kristensen et al., 2024 [51]											Low risk 8
Lang et al., 2021 [52]											Moderate risk 7
Laursen et al., 2021 [53]											Moderate risk 6
Lee et al., 2020 [54]											Low risk 10
Lei et al., 2022 [55]											Low risk 9
Leroux et al., 2015 [56]											Moderate risk 7
Lin et al., 2019 [57]											Moderate risk 6
Li et al., 2022 [58]											Moderate risk 6
Mardas et al., 2015 [59]											Moderate risk 6
Mardas et al., 2016 [60]											Moderate risk 6
Mazzeo et al., 2016 [61]											Moderate risk 6
Mehta et al., 2023 [62]											Low risk 8
Milajerdi et al., 2022 [63]											Moderate risk 6
Morton et al., 2020 [64]											Moderate risk 6
Na et al., 2021 [65]											Low risk 9
Petrick et al., 2015 [66]											Moderate risk 7
Piotrowicz et al., 2015 [67]											Moderate risk 7
Polderman et al., 2021 [68]											Moderate risk 7
Rej et al., 2021 [69]											Low risk 9
Shin et al., 2016 [70]											Moderate risk 6
Shi et al., 2020 [71]											Low risk 9
Shu et al., 2017 [72]											Moderate risk 7
Silveira et al., 2021 [73]											Low risk 8
Silviera et al., 2025 [74]											Moderate risk 6
Smith et al., 2024 [75]											Moderate risk 7
Subih et al., 2023 [76]											Moderate risk 7
Taha et al., 2022 [77]											Moderate risk 7
Tanaka et al., 2020 [78]											Moderate risk 7
Tasson et al., 2017 [79]											Moderate risk 6
Teasdale et al., 2020 [80]											Moderate risk 6
Tedeschi et al., 2017 [81]											Low risk 9
Thewjitcharoen et al., 2018 [82]											Moderate risk 6
Thomson et al., 2024 [83]											Low risk 9
Tseng et al., 2022 [84]											Moderate risk 7
Van Blarigan et al., 2020 [85]											Moderate risk 8
Van Lanen et al., 2024 [86]											Low risk 9
Wu et al., 2021 [87]											Low risk 8
Zeng et al., 2017 [88]											Low risk 10

Legend: Low risk of bias: 8-10/10; Moderate risk of bias: 5-7/10; High risk of bias: 0-4/10; Orange represents information is unclear, red is not stated / included, green is included

Domain items explanation from Hoy et al [1]

External validity assessment

Item 1 Was the study's target population **a close representation** of the national population in relation to relevant variables, e.g. age, sex, occupation?

Item 2 Was the sampling frame a **true or close representation** of the target population?

Item 3 Was some form of **random selection** used to select the sample, OR, was a census undertaken?

Item 4 Was the likelihood of **non-response bias minimal?**

Internal validity assessment

Item 5 Were data collected **directly from the subjects** (as opposed to a proxy)?

Item 6 Was an acceptable case definition used in the study?

Item 7 Was the study instrument that measured the parameter of interest (e.g. prevalence of low back pain) shown to have **reliability and validity (if necessary)**?

Item 8 Was the **same mode of data collection** used for all subjects?

Item 9 Was the **length of the shortest prevalence period** for the parameter of interest appropriate?

Item 10 Were the **numerator(s) and denominator(s)** for the parameter of interest appropriate?

References:

1. Hoy, D., P. Brooks, A. Woolf, F. Blyth, L. March, C. Bain, P. Baker, E. Smith and R. Buchbinder. "Assessing risk of bias in prevalence studies: Modification of an existing tool and evidence of interrater agreement." *J Clin Epidemiol* 65 (2012): 934-9. 10.1016/j.jclinepi.2011.11.014.
2. Adanan, N. I. H., M. S. Md Ali, J. H. Lim, N. F. Zakaria, C. T. S. Lim, R. Yahya, A. H. Abdul Gafor, T. Karupaiah and Z. M. Daud. "Investigating physical and nutritional changes during prolonged intermittent fasting in hemodialysis patients: A prospective cohort study." *Journal of Renal Nutrition* 30 (2020): e15-e26. 10.1053/j.jrn.2019.06.003. https://www.scopus.com/inward/record.uri?eid=2-s2.0-85070522588&doi=10.1053%2fj.jrn.2019.06.003&partnerID=40&md5=4514229e68d6f6f501d1776edfcc6bfc.
3. Affret, A., S. Wagner, D. El Fatouhi, C. Dow, E. Correia, M. Niravong, F. Clavel-Chapelon, J. De Chefdebien, D. Fouque, B. Stengel*, et al.* "Validity and reproducibility of a short food frequency questionnaire among patients with chronic kidney disease." *BMC Nephrology* 18 (2017): 10.1186/s12882-017-0695-2. https://www.scopus.com/inward/record.uri?eid=2-s2.0-85029518222&doi=10.1186%2fs12882-017-0695-2&partnerID=40&md5=ff51223d279ac6ac4149d0e0be498265.
4. Ahola, A. J., C. Forsblom and P. H. Groop. "Adherence to special diets and its association with meeting the nutrient recommendations in individuals with type 1 diabetes." *Acta Diabetologica* 55 (2018): 843-51. 10.1007/s00592-018-1159-2. https://www.scopus.com/inward/record.uri?eid=2-s2.0-85047161885&doi=10.1007%2fs00592-018-1159-2&partnerID=40&md5=7d56545d0cf248b955e2925d9fb95594.
5. Ahola, A. J., C. M. Forsblom, V. Harjutsalo and P. H. Groop. "Nut consumption is associated with lower risk of metabolic syndrome and its components in type 1 diabetes." *Nutrients* 13 (2021): 10.3390/nu13113909. https://www.scopus.com/inward/record.uri?eid=2-s2.0-

85118372100&doi=10.3390%2fnu13113909&partnerID=40&md5=4363363a131eeceab14de5543050fad3.
6. Amalia, R. I. and A. Davenport. "Estimated dietary sodium intake in peritoneal dialysis patients using food frequency questionnaires and total urinary and peritoneal sodium losses and assessment of extracellular volumes." *European Journal of Clinical Nutrition* 73 (2019): 105-11. 10.1038/s41430-018-0259-y. https://www.scopus.com/inward/record.uri?eid=2-s2.0-85050692907&doi=10.1038%2fs41430-018-0259-y&partnerID=40&md5=d91a5bbb7f0941ff43b7ab61ef05d41a.
7. Aponte, C. A. and R. G. Romanczyk. "Assessment of feeding problems in children with autism spectrum disorder." *Research in Autism Spectrum Disorders* 21 (2016): 61-72. 10.1016/j.rasd.2015.09.007. https://www.scopus.com/inward/record.uri?eid=2-s2.0-84945190001&doi=10.1016%2fj.rasd.2015.09.007&partnerID=40&md5=2f9dab46eec7debd205d882af025dd2c.
8. Arthur, A. E., A. M. Goss, W. Demark-Wahnefried, A. M. Mondul, K. R. Fontaine, Y. T. Chen, W. R. Carroll, S. A. Spencer, L. Q. Rogers, L. S. Rozek*, et al.* "Higher carbohydrate intake is associated with increased risk of all-cause and disease-specific mortality in head and neck cancer patients: Results from a prospective cohort study." *Int J Cancer* 143 (2018): 1105-13. 10.1002/ijc.31413.
9. Bail, J. R., S. V. Bail, J. Cagle, K. Tiesi, J. Caffey, M. Bakitas and W. Demark-Wahnefried. "Health behaviors and well-being among those “living” with metastatic cancer in alabama." *Supportive Care in Cancer* 30 (2022): 1689-701. 10.1007/s00520-021-06583-1. https://www.scopus.com/inward/record.uri?eid=2-s2.0-85115703930&doi=10.1007%2fs00520-021-06583-1&partnerID=40&md5=957457c5584055976cb6808889e9b92c.
10. Baleato, C. L., J. J. A. Ferguson, C. Oldmeadow, G. D. Mishra and M. L. Garg. "Plant-based dietary patterns versus meat consumption and prevalence of impaired glucose intolerance and diabetes mellitus: A cross-sectional study in australian women." *Nutrients* 14 (2022): 10.3390/nu14194152. https://www.scopus.com/inward/record.uri?eid=2-s2.0-85139858951&doi=10.3390%2fnu14194152&partnerID=40&md5=f412e6ab59bbb999458732225f162f2c.
11. Basu, A., A. C. Alman and J. K. Snell-Bergeon. "Dietary fiber intake and glycemic control: Coronary artery calcification in type 1 diabetes (cacti) study." *Nutrition Journal* 18 (2019): 10.1186/s12937-019-0449-z. https://www.scopus.com/inward/record.uri?eid=2-s2.0-85063960933&doi=10.1186%2fs12937-019-0449-z&partnerID=40&md5=1b3f35a1d8ad86e062d41118b6d499f4.
12. Basu, A., A. C. Alman and J. K. Snell-Bergeon. "Associations of dietary patterns and nutrients with glycated hemoglobin in participants with and without type 1 diabetes." *Nutrients* 13 (2021): 10.3390/nu13031035. https://www.scopus.com/inward/record.uri?eid=2-s2.0-85102814933&doi=10.3390%2fnu13031035&partnerID=40&md5=db7811b096c2a5173d86cbb0dab25fbb.
13. Beeren, I., L. de Goeij, R. Dandis, N. Vidra, M. van Zutphen, J. A. Witjes, E. Kampman, L. A. L. M. Kiemeney and A. Vrieling. "Limited changes in lifestyle behaviours after non-muscle invasive bladder cancer diagnosis." *Cancers* 14 (2022): 10.3390/cancers14040960. https://www.scopus.com/inward/record.uri?eid=2-s2.0-85124456153&doi=10.3390%2fcancers14040960&partnerID=40&md5=d54bb0c8ced060ada3bf6dab4b760a81.
14. Beiner, C., M. M. Qureshi, J. Zhao, B. Hu, R. Jimenez and A. E. Hirsch. "Depression and anxiety among english- and spanish-speaking patients with breast cancer receiving radiation therapy." *Int J Radiat Oncol Biol Phys* 119 (2024): 185-92. 10.1016/j.ijrobp.2023.11.049.
15. Belle, F. N., A. Chatelan, R. Kasteler, L. Mader, I. Guessous, M. Beck-Popovic, M. Ansari, C. E. Kuehni and M. Bochud. "Dietary intake and diet quality of adult survivors of childhood cancer and the general population: Results from the sccss-nutrition study." *Nutrients* 13 (2021): 10.3390/nu13061767. https://www.scopus.com/inward/record.uri?eid=2-s2.0-

85106310993&doi=10.3390%2fnu13061767&partnerID=40&md5=42591e4934dcb02341ecc8189478d363.
16. Birketvedt, K., A. Mikkelsen, L. L. Klingen, C. Henriksen, I. B. Helland and R. Emblem. "Nutritional status in adolescents with esophageal atresia." *J Pediatr* 218 (2020): 130-37. 10.1016/j.jpeds.2019.11.034.
17. Black, L. J., S. Hetherton, M. Forkan, E. G. Gonzales, J. B. Smith, A. Daly, R. M. Lucas and A. Langer-Gould. "An exploratory study of diet in childhood and young adulthood and adult-onset multiple sclerosis." *Multiple Sclerosis Journal* 27 (2021): 1611-14. 10.1177/1352458520986964. https://www.scopus.com/inward/record.uri?eid=2-s2.0-85099705009&doi=10.1177%2f1352458520986964&partnerID=40&md5=41a10ab8a0cd646123a3a5d0aedd28b5.
18. Bolte, L. A., K. A. Lee, J. R. Björk, E. R. Leeming, M. J. E. Campmans-Kuijpers, J. J. De Haan, A. V. Vila, A. Maltez-Thomas, N. Segata, R. Board*, et al.* "Association of a mediterranean diet with outcomes for patients treated with immune checkpoint blockade for advanced melanoma." *JAMA Oncology* 9 (2023): 705-09. 10.1001/jamaoncol.2022.7753. https://www.scopus.com/inward/record.uri?eid=2-s2.0-85159765905&doi=10.1001%2fjamaoncol.2022.7753&partnerID=40&md5=f8a59f8e0349fdd2335b6c6d9928da7d.
19. Boucher, B. A., S. Wanigaratne, S. A. Harris and M. Cotterchio. "Postdiagnosis isoflavone and lignan intake in newly diagnosed breast cancer patients: Cross-sectional survey shows considerable intake from previously unassessed high-lignan foods." *Current Developments in Nutrition* 2 (2018): https://www.scopus.com/inward/record.uri?eid=2-s2.0-85063312014&partnerID=40&md5=82c2487e03c9dbe190ed2755b0e2807a.
20. Bredin, C., S. Naimimohasses, S. Norris, C. Wright, N. Hancock, K. Hart and J. B. Moore. "Development and relative validation of a short food frequency questionnaire for assessing dietary intakes of non-alcoholic fatty liver disease patients." *European Journal of Nutrition* 59 (2020): 571-80. 10.1007/s00394-019-01926-5. https://www.scopus.com/inward/record.uri?eid=2-s2.0-85062148721&doi=10.1007%2fs00394-019-01926-5&partnerID=40&md5=97d511cc7089fe8f821c8e118f9b2024.
21. Chhabra, R. and A. Davenport. "Is increased subjective thirst associated with greater interdialytic weight gains, extracellular fluid and dietary sodium intake?" *Artificial Organs* 48 (2024): 91-97. 10.1111/aor.14657. https://www.scopus.com/inward/record.uri?eid=2-s2.0-85175378892&doi=10.1111%2faor.14657&partnerID=40&md5=5dadde7bc97bd1e7aebac2d985428559.
22. Coe, S., S. L. Spruzen, C. Sanchez, H. Izadi and H. Dawes. "A cross-sectional feasibility study of nutrient intake patterns in people with parkinson's compared to government nutrition guidelines." *J Am Coll Nutr* 39 (2020): 187-91. 10.1080/07315724.2019.1633440.
23. Conley, M., K. L. Campbell, C. M. Hawley, N. M. Lioufas, G. J. Elder, S. V. Badve, E. Pedagogos, E. Milanzi, E. M. Pascoe, A. Valks*, et al.* "Relationship between dietary phosphate intake and biomarkers of bone and mineral metabolism in australian adults with chronic kidney disease." *J Ren Nutr* 32 (2022): 58-67. 10.1053/j.jrn.2021.07.004.
24. Cooke, Z. M., S. M. Resciniti, B. J. Wright, M. W. Hale, C. K. Yao, C. J. Tuck and J. R. Biesiekierski. "Association between dietary factors, symptoms, and psychological factors in adults with dyspepsia: A cross-sectional study." *Neurogastroenterology and Motility* 35 (2023): 10.1111/nmo.14684. https://www.scopus.com/inward/record.uri?eid=2-s2.0-85173553019&doi=10.1111%2fnmo.14684&partnerID=40&md5=ffe9ead908e24afe527e372dc05b1b79.
25. Crowder, S. L., Z. Li, K. P. Sarma and A. E. Arthur. "Chronic nutrition impact symptoms are associated with decreased functional status, quality of life, and diet quality in a pilot study of long-term post-radiation head and neck cancer survivors." *Nutrients* 13 (2021): 10.3390/nu13082886. https://www.scopus.com/inward/record.uri?eid=2-s2.0-85113806378&doi=10.3390%2fnu13082886&partnerID=40&md5=8fc2c54be99e383e8c4d27102ac9e279.

26. Dewinter, L., K. Casteels, K. Corthouts, K. Van De Kerckhove, K. Van Der Vaerent, K. Vanmeerbeeck and C. Matthys. "Dietary intake of non-nutritive sweeteners in type 1 diabetes mellitus children." *Food Additives and Contaminants - Part A Chemistry, Analysis, Control, Exposure and Risk Assessment* 33 (2015): 19-26. 10.1080/19440049.2015.1112039. https://www.scopus.com/inward/record.uri?eid=2-s2.0-84946935036&doi=10.1080%2f19440049.2015.1112039&partnerID=40&md5=d590a88a02968edb20aafc7d2c0e3e76.
27. Dinparast, F., A. Sharifi, S. Moradi, M. Alipour and B. Alipour. "The associations between dietary pattern of chronic obstructive pulmonary disease patients and depression: A cross-sectional study." *BMC Pulmonary Medicine* 21 (2021): 10.1186/s12890-020-01383-5. https://www.scopus.com/inward/record.uri?eid=2-s2.0-85098796061&doi=10.1186%2fs12890-020-01383-5&partnerID=40&md5=2ec7546e839acd2c117ecb3c7c2004aa.
28. Dolovich, C., L. A. Shafer, K. Vagianos, K. Witges, L. E. Targownik and C. N. Bernstein. "The complex relationship between diet, symptoms, and intestinal inflammation in persons with inflammatory bowel disease: The manitoba living with ibd study." *Journal of Parenteral and Enteral Nutrition* 46 (2022): 867-77. 10.1002/jpen.2257. https://www.scopus.com/inward/record.uri?eid=2-s2.0-85115306889&doi=10.1002%2fjpen.2257&partnerID=40&md5=bf1be726cd2fbb3eba49465515f6dab7.
29. Dratsky, D., E. McGillivray, J. Mittal, E. A. Handorf, G. Berardi, I. Astsaturov, M. J. Hall, M. C. Yeh, R. Jain and C. Y. Fang. "Food insecurity and dietary quality in african american patients with gastrointestinal cancers: An exploratory study." *Nutrients* 16 (2024): 10.3390/nu16183057.
30. Drzymała-Czyz, S., Ł. Kałuzny, P. Krzyzanowska-Jankowska, D. Walkowiak, R. Mozrzymas and J. Walkowiak. "Deficiency of long-chain polyunsaturated fatty acids in phenylketonuria: A cross-sectional study." *Acta Biochimica Polonica* 65 (2018): 303-08. 10.18388/abp.2018_2565. https://www.scopus.com/inward/record.uri?eid=2-s2.0-85048697024&doi=10.18388%2fabp.2018_2565&partnerID=40&md5=84f141ab0f8748c0fb496cb6bc1221b3.
31. Ericson, J., L. Lundell, M. Lindblad, F. Klevebro, M. Nilsson and I. Rouvelas. "Assessment of energy intake and total energy expenditure in a series of patients who have undergone oesophagectomy following neoadjuvant treatment." *Clinical Nutrition ESPEN* 37 (2020): 121-28. 10.1016/j.clnesp.2020.03.007. https://www.scopus.com/inward/record.uri?eid=2-s2.0-85082826754&doi=10.1016%2fj.clnesp.2020.03.007&partnerID=40&md5=18399c5b053a7d80109ffb8954346290.
32. Ewers, B., E. Trolle, S. S. Jacobsen, D. Vististen, T. P. Almdal, T. Vilsbøll and J. M. Bruun. "Dietary habits and adherence to dietary recommendations in patients with type 1 and type 2 diabetes compared with the general population in denmark." *Nutrition* 61 (2019): 49-55. 10.1016/j.nut.2018.10.021. https://www.scopus.com/inward/record.uri?eid=2-s2.0-85060336949&doi=10.1016%2fj.nut.2018.10.021&partnerID=40&md5=b4da7a7a81f1a3098ea0e83e15cc09ec.
33. Ferrari, A., A. M. de Carvalho, J. Steluti, J. Teixeira, D. M. L. Marchioni and S. Aguiar. "Folate and nutrients involved in the 1-carbon cycle in the pretreatment of patients for colorectal cancer." *Nutrients* 7 (2015): 4318-35. 10.3390/nu7064318. https://www.scopus.com/inward/record.uri?eid=2-s2.0-84931281789&doi=10.3390%2fnu7064318&partnerID=40&md5=0b654cf756d6243d90753a5424225b87.
34. Fisher, E. L., N. A. Weaver, A. L. Marlow, B. R. King and C. E. Smart. "Macronutrient intake in children and adolescents with type 1 diabetes and its association with glycemic outcomes." *Pediatric Diabetes* 2023 (2023): 10.1155/2023/7102890. https://www.scopus.com/inward/record.uri?eid=2-s2.0-85179099799&doi=10.1155%2f2023%2f7102890&partnerID=40&md5=a9e119ae304c59dd6c89beb0fdafc344.

35. Ganguzza, L., C. Ngai, L. Flink, K. Woolf, Y. Guo, E. Gianos, J. Burdowski, J. Slater, V. Acosta, T. Shephard, *et al.* "Association between diet quality and measures of body adiposity using the rate your plate survey in patients presenting for coronary angiography." *Clin Cardiol* 41 (2018): 126-30. 10.1002/clc.22843.
36. Gilbertson, H. R., K. Reed, S. Clark, K. L. Francis and F. J. Cameron. "An audit of the dietary intake of australian children with type 1 diabetes." *Nutrition and Diabetes* 8 (2018): 10.1038/s41387-018-0021-5. https://www.scopus.com/inward/record.uri?eid=2-s2.0-85044226999&doi=10.1038%2fs41387-018-0021-5&partnerID=40&md5=2feb30ee0287c7754d12f1039ade0409.
37. Gingras, V., C. Leroux, K. Desjardins, V. Savard, S. Lemieux, R. Rabasa-Lhoret and I. Strychar. "Association between cardiometabolic profile and dietary characteristics among adults with type 1 diabetes mellitus." *J Acad Nutr Diet* 115 (2015): 1965-74. 10.1016/j.jand.2015.04.012.
38. Godny, L., N. Maharshak, L. Reshef, I. Goren, L. Yahav, N. Fliss-Isakov, U. Gophna, H. Tulchinsky and I. Dotan. "Fruit consumption is associated with alterations in microbial composition and lower rates of pouchitis." *Journal of Crohn's and Colitis* 13 (2019): 1265-72. 10.1093/ecco-jcc/jjz053. https://www.scopus.com/inward/record.uri?eid=2-s2.0-85067186376&doi=10.1093%2fecco-jcc%2fjjz053&partnerID=40&md5=63780dd93af8936124693ffee18b42f9.
39. Gregg, J. R., J. Zheng, D. S. Lopez, C. Reichard, G. Browman, B. Chapin, J. Kim, J. Davis and C. R. Daniel. "Diet quality and gleason grade progression among localised prostate cancer patients on active surveillance." *British Journal of Cancer* 120 (2019): 466-71. 10.1038/s41416-019-0380-2. https://www.scopus.com/inward/record.uri?eid=2-s2.0-85060732952&doi=10.1038%2fs41416-019-0380-2&partnerID=40&md5=7ea60b73db5203737781c99c3bd057bf.
40. Grieco, L. P., T. M. Brasky, C. K. Spees and J. L. Krok-Schoen. "The associations between dietary supplement use, diet quality, and health-related quality of life among older female cancer survivors." *Nutrition and Cancer* 74 (2022): 2829-37. 10.1080/01635581.2022.2035779. https://www.scopus.com/inward/record.uri?eid=2-s2.0-85124256193&doi=10.1080%2f01635581.2022.2035779&partnerID=40&md5=a55c8d97a3052ec0020c8c9089134f32.
41. Helm, M. M., A. Basu, L. A. Richardson, L. C. Chien, K. Izuora, A. C. Alman and J. K. Snell-Bergeon. "Longitudinal three-year associations of dietary fruit and vegetable intake with serum hs-c-reactive protein in adults with and without type 1 diabetes." *Nutrients* 16 (2024): 10.3390/nu16132058. https://www.scopus.com/inward/record.uri?eid=2-s2.0-85198347029&doi=10.3390%2fnu16132058&partnerID=40&md5=e902dcae5e5b8b5fae0adaaa37617c64.
42. Horikawa, C., K. Tsuda, Y. Oshida, J. Satoh, Y. Hayashino, N. Tajima, R. Nishimura, H. Sone, D. Koya, K. Shikata, *et al.* "Dietary intake and physical activity in japanese patients with type 2 diabetes: The japan diabetes complication and its prevention prospective study (jdcp study 8)." *Diabetology International* 13 (2022): 344-57. 10.1007/s13340-022-00575-0. https://www.scopus.com/inward/record.uri?eid=2-s2.0-85132614435&doi=10.1007%2fs13340-022-00575-0&partnerID=40&md5=183491af63a7473b7e80ea3ecf144cb1.
43. Hu, J., C. La Vecchia, E. Negri, M. de Groh, H. Morrison and L. Mery. "Macronutrient intake and stomach cancer." *Cancer Causes and Control* 26 (2015): 839-47. 10.1007/s10552-015-0557-9. https://www.scopus.com/inward/record.uri?eid=2-s2.0-84929711905&doi=10.1007%2fs10552-015-0557-9&partnerID=40&md5=93a8df4e9d8f6adf6ff6f12b7972d75f.
44. Hussain, S. K., T. S. Dong, V. Agopian, J. R. Pisegna, F. A. Durazo, P. Enayati, V. Sundaram, J. N. Benhammou, M. Noureddin, G. Choi, *et al.* "Dietary protein, fiber and coffee are associated with small intestine microbiome composition and diversity in patients with liver cirrhosis." *Nutrients* 12 (2020): 10.3390/nu12051395. https://www.scopus.com/inward/record.uri?eid=2-s2.0-

85084786589&doi=10.3390%2fnu12051395&partnerID=40&md5=b65cd21c620789951238be3e7a268f8a.

45. Ijpma, I., R. J. Renken, J. A. Gietema, R. H. J. A. Slart, M. G. J. Mensink, J. D. Lefrandt, G. J. Ter Horst and A. K. L. Reyners. "Changes in taste and smell function, dietary intake, food preference, and body composition in testicular cancer patients treated with cisplatin-based chemotherapy." *Clinical Nutrition* 36 (2017): 1642-48. 10.1016/j.clnu.2016.10.013. https://www.scopus.com/inward/record.uri?eid=2-s2.0-85006265392&doi=10.1016%2fj.clnu.2016.10.013&partnerID=40&md5=6d5538dc481a9154a2641d6d488a8cd9.

46. Ilari, S., L. Vitiello, P. Russo, S. Proietti, M. Milić, C. Muscoli, V. Cardaci, C. Tomino, G. Bonassi and S. Bonassi. "Daily vegetables intake and response to copd rehabilitation. The role of oxidative stress, inflammation and dna damage." *Nutrients* 13 (2021): 10.3390/nu13082787. https://www.scopus.com/inward/record.uri?eid=2-s2.0-85112348619&doi=10.3390%2fnu13082787&partnerID=40&md5=aacb937bde799f5d13fe27f5464de563.

47. Khatun, T., A. Hoque, K. S. Anwar, M. R. Sarker, F. Ara and D. Maqbool. "Dietary habits of patients with coronary artery disease in a tertiary-care hospital of bangladesh: A case-controlled study." *Journal of Health, Population and Nutrition* 40 (2021): 10.1186/s41043-021-00226-1. https://www.scopus.com/inward/record.uri?eid=2-s2.0-85101838019&doi=10.1186%2fs41043-021-00226-1&partnerID=40&md5=1f2fc387db784e76cf1a68d8c53b6296.

48. Kiew, S. J., N. A. Mohd Taib, T. Islam and H. Abdul Majid. "Changes in dietary intake of breast cancer survivors: Early findings of a malaysian breast cancer prospective cohort study." *Nutrition and Cancer* 74 (2022): 2470-78. 10.1080/01635581.2021.2013508. https://www.scopus.com/inward/record.uri?eid=2-s2.0-85121450000&doi=10.1080%2f01635581.2021.2013508&partnerID=40&md5=cbfc0b10efd7f748f1f7ef6c26219bbd.

49. Klimek, A., C. Baerwald, M. Schwarz, F. Rutsch, K. G. Parhofer, U. Plöckinger, M. Heddrich-Ellerbrok, S. Vom Dahl, K. Schöne, M. Ott*, et al.* "Everyday life, dietary practices, and health conditions of adult pku patients: A multicenter, cross-sectional study." *Ann Nutr Metab* 76 (2020): 251-58. 10.1159/000510260.

50. Knoerl, R., R. Ploutz-Snyder, L. Smener, C. Tofthagen and S. Zick. "Association of chemotherapy-induced peripheral neuropathy with diet quality among post-treatment cancer survivors." *Nutrition and Cancer* 76 (2024): 717-25. 10.1080/01635581.2024.2364389. https://www.scopus.com/inward/record.uri?eid=2-s2.0-85197216201&doi=10.1080%2f01635581.2024.2364389&partnerID=40&md5=a388c3a507287e4bed12984d78e55d36.

51. Kristensen, M. B., C. L. Egholm, H. S. Vistisen, B. Borregaard, S. M. Bruvik, B. M. Bertelsen, E. Myrup, T. Mortensen, L. Viggers, R. E. Mols*, et al.* "Challenges and benefits of using the heartdiet food frequency questionnaire in cardiac rehabilitation practice." *Nutrition, Metabolism and Cardiovascular Diseases* 34 (2024): 1968-75. 10.1016/j.numecd.2024.04.016. https://www.scopus.com/inward/record.uri?eid=2-s2.0-85195645098&doi=10.1016%2fj.numecd.2024.04.016&partnerID=40&md5=61e1ad494167bad66cc60f640c2a96c7.

52. Lang, S., A. Martin, X. Zhang, F. Farowski, H. Wisplinghoff, M. J.G.T. Vehreschild, M. Krawczyk, A. Nowag, A. Kretzschmar, C. Scholz*, et al.* "Combined analysis of gut microbiota, diet and pnpla3 polymorphism in biopsy-proven non-alcoholic fatty liver disease." *Liver International* 41 (2021): 1576-91. 10.1111/liv.14899. https://www.scopus.com/inward/record.uri?eid=2-s2.0-85105243459&doi=10.1111%2fliv.14899&partnerID=40&md5=e4365f4ce77783c40aa4d91b1174958f.

53. Laursen, U. B., M. N. Johansen, A. M. Joensen, K. Overvad and M. L. Larsen. "Is cardiac rehabilitation equally effective in improving dietary intake in all patients with ischemic heart disease?" *Journal of the American College of Nutrition* 40 (2021): 33-40. 10.1080/07315724.2020.1755910. https://www.scopus.com/inward/record.uri?eid=2-s2.0-

85086368945&doi=10.1080%2f07315724.2020.1755910&partnerID=40&md5=8d0454d0b17ee9352939462c876e5d44.

54. Lee, H., H. Kim, T. Y. Kim, H. Ryu, D. L. Ju, M. Jang, K. H. Oh, C. Ahn and S. N. Han. "Dietary assessment of korean non-dialysis chronic kidney disease patients with or without diabetes." *J Korean Med Sci* 35 (2020): e181. 10.3346/jkms.2020.35.e181.
55. Lei, Y. Y., S. C. Ho, C. Kwok, A. Cheng, K. L. Cheung, R. Lee, F. K. F. Mo and W. Yeo. "Association of high adherence to vegetables and fruits dietary pattern with quality of life among chinese women with early-stage breast cancer." *Quality of Life Research* 31 (2022): 1371-84. 10.1007/s11136-021-02985-0. https://www.scopus.com/inward/record.uri?eid=2-s2.0-85115099134&doi=10.1007%2fs11136-021-02985-0&partnerID=40&md5=5a85e9f858bda753bb2e224a90c9bbb2.
56. Leroux, C., V. Gingras, K. Desjardins, A. S. Brazeau, S. Ott-Braschi, I. Strychar and R. Rabasa-Lhoret. "In adult patients with type 1 diabetes healthy lifestyle associates with a better cardiometabolic profile." *Nutr Metab Cardiovasc Dis* 25 (2015): 444-51. 10.1016/j.numecd.2015.01.004.
57. Lin, I. H., T. C. Wong, S. W. Nien, Y. T. Chou, Y. J. Chiang, H. H. Wang and S. H. Yang. "Dietary compliance among renal transplant recipients: A single-center study in taiwan." *Transplant Proc* 51 (2019): 1325-30. 10.1016/j.transproceed.2019.02.026.
58. Li, Q. H., Y. W. Zou, S. Y. Lian, J. J. Liang, Y. F. Bi, C. Deng, Y. Q. Mo, K. M. Yang and L. Dai. "Sugar-sweeten beverage consumption is associated with more obesity and higher serum uric acid in chinese male gout patients with early onset." *Frontiers in Nutrition* 9 (2022): 10.3389/fnut.2022.916811. https://www.scopus.com/inward/record.uri?eid=2-s2.0-85134926611&doi=10.3389%2ffnut.2022.916811&partnerID=40&md5=3ac19c665c85bdc50097f236595664e3.
59. Mardas, M., M. Jamka, R. Mądry, J. Walkowiak, M. Krótkopad and M. Stelmach-Mardas. "Dietary habits changes and quality of life in patients undergoing chemotherapy for epithelial ovarian cancer." *Supportive Care in Cancer* 23 (2015): 1015-23. 10.1007/s00520-014-2462-2. https://www.scopus.com/inward/record.uri?eid=2-s2.0-84930509570&doi=10.1007%2fs00520-014-2462-2&partnerID=40&md5=f6b15d39b30a1269ca45f7f7879b1667.
60. Mardas, M., R. Mądry and M. Stelmach-Mardas. "Dietary intake variability in the cycle of cytotoxic chemotherapy." *Supportive Care in Cancer* 24 (2016): 2619-25. 10.1007/s00520-015-3072-3. https://www.scopus.com/inward/record.uri?eid=2-s2.0-84953258159&doi=10.1007%2fs00520-015-3072-3&partnerID=40&md5=6572c550a279f324687da8cf2bc30150.
61. Mazzeo, T., L. Roncoroni, V. Lombardo, C. Tomba, L. Elli, S. Sieri, S. Grioni, M. T. Bardella, C. Agostoni, L. Doneda*, et al.* "Evaluation of a modified italian european prospective investigation into cancer and nutrition food frequency questionnaire for individuals with celiac disease." *Journal of the Academy of Nutrition and Dietetics* 116 (2016): 1810-16. 10.1016/j.jand.2016.04.013. https://www.scopus.com/inward/record.uri?eid=2-s2.0-84969983892&doi=10.1016%2fj.jand.2016.04.013&partnerID=40&md5=611bcc3de7bc870c28f6b40c11531bdd.
62. Mehta, P., Q. Li, M. Stahl, U. Uusitalo, K. Lindfors, M. D. Butterworth, K. Kurppa, S. Virtanen, S. Koletzko, C. Aronsson*, et al.* "Gluten-free diet adherence in children with screening-detected celiac disease using a prospective birth cohort study." *PLoS ONE* 18 (2023): 10.1371/journal.pone.0275123. https://www.scopus.com/inward/record.uri?eid=2-s2.0-85147318953&doi=10.1371%2fjournal.pone.0275123&partnerID=40&md5=b3881fcef6496461ff5f7a2eefe97ecf.
63. Milajerdi, A., M. Shayanfar, S. Benisi-Kohansal, M. Mohammad-Shirazi, G. Sharifi, H. Tabibi and A. Esmaillzadeh. "A case-control study on dietary acid load in relation to glioma." *Nutrition and Cancer* 74 (2022): 1644-51. 10.1080/01635581.2021.1957134. https://www.scopus.com/inward/record.uri?eid=2-s2.0-

85111690191&doi=10.1080%2f01635581.2021.1957134&partnerID=40&md5=f062133501000dd1fb80dd80e07cbb54.
64. Morton, H., K. C. Pedley, R. J. C. Stewart and J. Coad. "Inflammatory bowel disease: Are symptoms and diet linked?" *Nutrients* 12 (2020): 1-14. 10.3390/nu12102975. https://www.scopus.com/inward/record.uri?eid=2-s2.0-85091788868&doi=10.3390%2fnu12102975&partnerID=40&md5=37771c730cd500c9ff11fef59438a672.
65. Na, W., Y. Lee, H. Kim, Y. S. Kim and C. Sohn. "High-fat foods and fodmaps containing gluten foods primarily contribute to symptoms of irritable bowel syndrome in korean adults." *Nutrients* 13 (2021): 10.3390/nu13041308. https://www.scopus.com/inward/record.uri?eid=2-s2.0-85104110431&doi=10.3390%2fnu13041308&partnerID=40&md5=09f272b8eb78fecf12ff47309af4440d.
66. Petrick, J. L., S. E. Steck, P. T. Bradshaw, W. H. Chow, L. S. Engel, K. He, H. A. Risch, T. L. Vaughan and M. D. Gammon. "Dietary flavonoid intake and barrett's esophagus in western washington state." *Annals of Epidemiology* 25 (2015): 730-35.e2. 10.1016/j.annepidem.2015.05.010. https://www.scopus.com/inward/record.uri?eid=2-s2.0-84941189532&doi=10.1016%2fj.annepidem.2015.05.010&partnerID=40&md5=3c7e82f30b532b680f66c4a7d0060878.
67. Piotrowicz, K., E. Pałkowska, E. Bartnikowska, P. Krzesiński, A. Stańczyk, P. Biecek, A. Skrobowski and G. Gielerak. "Self-reported health-related behaviors and dietary habits in patients with metabolic syndrome." *Cardiology Journal* 22 (2015): 413-20. 10.5603/CJ.a2015.0020. https://www.scopus.com/inward/record.uri?eid=2-s2.0-84940530640&doi=10.5603%2fCJ.a2015.0020&partnerID=40&md5=8f5729990d9dafea184ffda66710c60a.
68. Polderman, N., M. Cushing, K. McFadyen, M. Catapang, R. Humphreys, C. Mammen, D. G. Matsell and T. Pediatric Nephrology Clinical Pathway Development. "Dietary intakes of children with nephrotic syndrome." *Pediatr Nephrol* 36 (2021): 2819-26. 10.1007/s00467-021-05055-2. https://www.ncbi.nlm.nih.gov/pubmed/33783623.
69. Rej, A., C. C. Shaw, R. L. Buckle, N. Trott, A. Agrawal, K. Mosey, K. Sanders, R. Allen, S. Martin, A. Newton*, et al.* "The low fodmap diet for ibs; a multicentre uk study assessing long term follow up." *Digestive and Liver Disease* 53 (2021): 1404-11. 10.1016/j.dld.2021.05.004. https://www.scopus.com/inward/record.uri?eid=2-s2.0-85107117027&doi=10.1016%2fj.dld.2021.05.004&partnerID=40&md5=43119ae2b1e871bdb688474ff42820f0.
70. Shin, W. K., S. Song, E. Hwang, H. G. Moon, D. Y. Noh and J. E. Lee. "Development of a ffq for breast cancer survivors in korea." *British Journal of Nutrition* 116 (2016): 1781-86. 10.1017/S000711451600372X. https://www.scopus.com/inward/record.uri?eid=2-s2.0-84995460834&doi=10.1017%2fS000711451600372X&partnerID=40&md5=25310da1fce3ff8b30aabd3a50d77ae0.
71. Shi, Z., A. Rundle, J. M. Genkinger, Y. K. Cheung, I. J. Ergas, J. M. Roh, L. H. Kushi, M. L. Kwan and H. Greenlee. "Distinct trajectories of fruits and vegetables, dietary fat, and alcohol intake following a breast cancer diagnosis: The pathways study." *Breast Cancer Research and Treatment* 179 (2020): 229-40. 10.1007/s10549-019-05457-9. https://www.scopus.com/inward/record.uri?eid=2-s2.0-85073953356&doi=10.1007%2fs10549-019-05457-9&partnerID=40&md5=f814f25639c8cca4e5abbea19590aeb3.
72. Shu, P. S., Y. M. Chan and S. L. Huang. "Higher body mass index and lower intake of dairy products predict poor glycaemic control among type 2 diabetes patients in malaysia." *PLoS ONE* 12 (2017): 10.1371/journal.pone.0172231. https://www.scopus.com/inward/record.uri?eid=2-s2.0-85014045164&doi=10.1371%2fjournal.pone.0172231&partnerID=40&md5=a254367c67d50b4d34065235377d7d95.
73. Silveira, S. L., B. Jeng, G. Cutter and R. W. Motl. "Diet quality assessment in wheelchair users with multiple sclerosis." *Nutrients* 13 (2021): 10.3390/nu13124352.

https://www.scopus.com/inward/record.uri?eid=2-s2.0-85120558037&doi=10.3390%2fnu13124352&partnerID=40&md5=40dadc1e8342ea52b3a85cf4fbeff4ce.

74. Silveira, S. L., B. Jeng, B. A. Gower, G. R. Cutter and R. W. Motl. "Correlates of inaccuracy in reporting of energy intake among persons with multiple sclerosis." *Nutrients* 17 (2025): 10.3390/nu17030438. https://www.scopus.com/inward/record.uri?eid=2-s2.0-85217786909&doi=10.3390%2fnu17030438&partnerID=40&md5=3f375f04a2737dd71c6225dc0a253447.

75. Smith, S., A. Fisher, P. J. Lally, H. A. Croker, A. Roberts, R. E. Conway and R. J. Beeken. "Perceiving a need for dietary change in adults living with and beyond cancer: A cross-sectional study." *Cancer Medicine* 13 (2024): 10.1002/cam4.7073. https://www.scopus.com/inward/record.uri?eid=2-s2.0-85187442479&doi=10.1002%2fcam4.7073&partnerID=40&md5=27503c37e32394c98f45fe00479bff0c.

76. Subih, H. S., E. A. Al-Shwaiyat, N. Al-Bayyari, B. S. Obeidat, F. Abu-Farsakh and H. Bawadi. "Dietary intake is not associated with body composition nor with biochemical tests but with psychological status of cancer patients receiving chemotherapy." *Nutrients* 15 (2023): 10.3390/nu15245087. https://www.scopus.com/inward/record.uri?eid=2-s2.0-85180688688&doi=10.3390%2fnu15245087&partnerID=40&md5=5ace61f2404c6cc347f480a848d646d4.

77. Taha, H. M., L. S. Rozek, X. Chen, Z. Li, K. R. Zarins, A. N. Slade, G. T. Wolf and A. E. Arthur. "Risk of disease recurrence and mortality varies by type of fat consumed before cancer treatment in a longitudinal cohort of head and neck squamous cell carcinoma patients." *Journal of Nutrition* 152 (2022): 1298-305. 10.1093/jn/nxac032. https://www.scopus.com/inward/record.uri?eid=2-s2.0-85129997700&doi=10.1093%2fjn%2fnxac032&partnerID=40&md5=e6bdcfd926f64fee501f1733dd3f123a.

78. Tanaka, Y., T. Nakagami, J. Oya, C. Ukita-Shibasaki, Y. Takehana, S. Sasaki and T. Babazono. "Impact of body mass index and age on the relative accuracy of self-reported energy intakes among japanese patients with type 2 diabetes." *Diabetology International* 11 (2020): 360-67. 10.1007/s13340-020-00430-0. https://www.scopus.com/inward/record.uri?eid=2-s2.0-85082808368&doi=10.1007%2fs13340-020-00430-0&partnerID=40&md5=d5e4bf77520487446ee125c13091d09d.

79. Tasson, L., C. Canova, M. G. Vettorato, E. Savarino and R. Zanotti. "Influence of diet on the course of inflammatory bowel disease." *Digestive Diseases and Sciences* 62 (2017): 2087-94. 10.1007/s10620-017-4620-0. https://www.scopus.com/inward/record.uri?eid=2-s2.0-85019710574&doi=10.1007%2fs10620-017-4620-0&partnerID=40&md5=441027a630a2d541c41991b0251dc5dd.

80. Teasdale, S. B., T. L. Burrows, T. Hayes, C. Y. Hsia, A. Watkins, J. Curtis and P. B. Ward. "Dietary intake, food addiction and nutrition knowledge in young people with mental illness." *Nutrition and Dietetics* 77 (2020): 315-22. 10.1111/1747-0080.12550. https://www.scopus.com/inward/record.uri?eid=2-s2.0-85068134009&doi=10.1111%2f1747-0080.12550&partnerID=40&md5=35e4b1557b2cd52b40fcf0dbf110ded8.

81. Tedeschi, S. K., M. Frits, J. Cui, Z. Z. Zhang, T. Mahmoud, C. Iannaccone, T. C. Lin, K. Yoshida, M. E. Weinblatt, N. A. Shadick*, et al.* "Diet and rheumatoid arthritis symptoms: Survey results from a rheumatoid arthritis registry." *Arthritis Care and Research* 69 (2017): 1920-25. 10.1002/acr.23225. https://www.scopus.com/inward/record.uri?eid=2-s2.0-85035070530&doi=10.1002%2facr.23225&partnerID=40&md5=f38c8139ed3c21c1310b0454b8256f02.

82. Thewjitcharoen, Y., P. Chotwanvirat, A. Jantawan, N. Siwasaranond, S. Saetung, H. Nimitphong, T. Himathongkam and S. Reutrakul. "Evaluation of dietary intakes and nutritional knowledge in thai patients with type 2 diabetes mellitus." *Journal of Diabetes Research* 2018 (2018): 10.1155/2018/9152910. https://www.scopus.com/inward/record.uri?eid=2-s2.0-

85060372932&doi=10.1155%2f2018%2f9152910&partnerID=40&md5=6040f1b2be3bd59c4c35f9a36e223224.
83. Thomson, R. L., J. D. Brown, H. Oakey, K. Palmer, P. Ashwood, M. A. S. Penno, K. J. McGorm, R. Battersby, P. G. Colman, M. E. Craig, *et al.* "Dietary patterns during pregnancy and maternal and birth outcomes in women with type 1 diabetes: The environmental determinants of islet autoimmunity (endia) study." *Diabetologia* 67 (2024): 2420-32. 10.1007/s00125-024-06259-5. https://www.scopus.com/inward/record.uri?eid=2-s2.0-85202966833&doi=10.1007%2fs00125-024-06259-5&partnerID=40&md5=8f195ac069211d81cd90eb72bc387c62.
84. Tseng, L. Y., W. Xie, W. Pan, H. Lyu, Z. Yu, W. Shi, Y. He, W. Chen, T. Li and E. Hsieh. "Validation of a six-item dietary calcium screening tool among hiv patients in china." *Public Health Nutrition* 24 (2021): 4786-95. 10.1017/S1368980021001427. https://www.scopus.com/inward/record.uri?eid=2-s2.0-85103757363&doi=10.1017%2fS1368980021001427&partnerID=40&md5=46191739f279eb30b5fd4d8eeba3cb33.
85. Van Blarigan, E. L., S. Zhang, F. S. Ou, A. Venlo, K. Ng, C. Atreya, K. Van Loon, D. Niedzwiecki, E. Giovannucci, E. G. Wolfe, *et al.* "Association of diet quality with survival among people with metastatic colorectal cancer in the cancer and leukemia b and southwest oncology group 80405 trial." *JAMA Network Open* 3 (2020): 10.1001/jamanetworkopen.2020.23500. https://www.scopus.com/inward/record.uri?eid=2-s2.0-85094935521&doi=10.1001%2fjamanetworkopen.2020.23500&partnerID=40&md5=3e74fa48a6f437d490fd4e8726daac16.
86. van Lanen, A. S., D. E. Kok, E. Wesselink, J. W. G. Derksen, A. M. May, K. C. Smit, M. Koopman, J. de Wilt, E. Kampman, F. J. B. van Duijnhoven, *et al.* "Associations between low- and high-fat dairy intake and recurrence risk in people with stage i–iii colorectal cancer differ by sex and primary tumour location." *International Journal of Cancer* 155 (2024): 828-38. 10.1002/ijc.34959. https://www.scopus.com/inward/record.uri?eid=2-s2.0-85192181875&doi=10.1002%2fijc.34959&partnerID=40&md5=4dbc9ce376113b72f1a050b8af7f596a.
87. Wu, W., M. J. L. Bours, A. Koole, M. F. Kenkhuis, S. J. P. M. Eussen, S. O. Breukink, F. J. van Schooten, M. P. Weijenberg and G. J. Hageman. "Cross-sectional associations between dietary daily nicotina-mide intake and patient-reported outcomes in colorectal cancer survivors, 2 to 10 years post-diagnosis." *Nutrients* 13 (2021): 10.3390/nu13113707. https://www.scopus.com/inward/record.uri?eid=2-s2.0-85117310592&doi=10.3390%2fnu13113707&partnerID=40&md5=f97a6c4ff19015540918cad394800e22.
88. Zeng, G., Z. Mai, S. Xia, Z. Wang, K. Zhang, L. Wang, Y. Long, J. Ma, Y. Li, S. P. Wan, *et al.* "Prevalence of kidney stones in china: An ultrasonography based cross-sectional study." *BJU Int* 120 (2017): 109-16. 10.1111/bju.13828.
