# Supplementary figures and images for: Completion Rates of Food Frequency Questionnaires and Food Records in People with Chronic Conditions: Systematic Review and Meta-Analysis
Source: Nutrients. 2026 Jun 13;18(12):1922. doi: 10.3390/nu18121922 (PMC13306072; doi:10.3390/nu18121922)

Supplementary Figure S1. Funnel plot of all eligible studies.

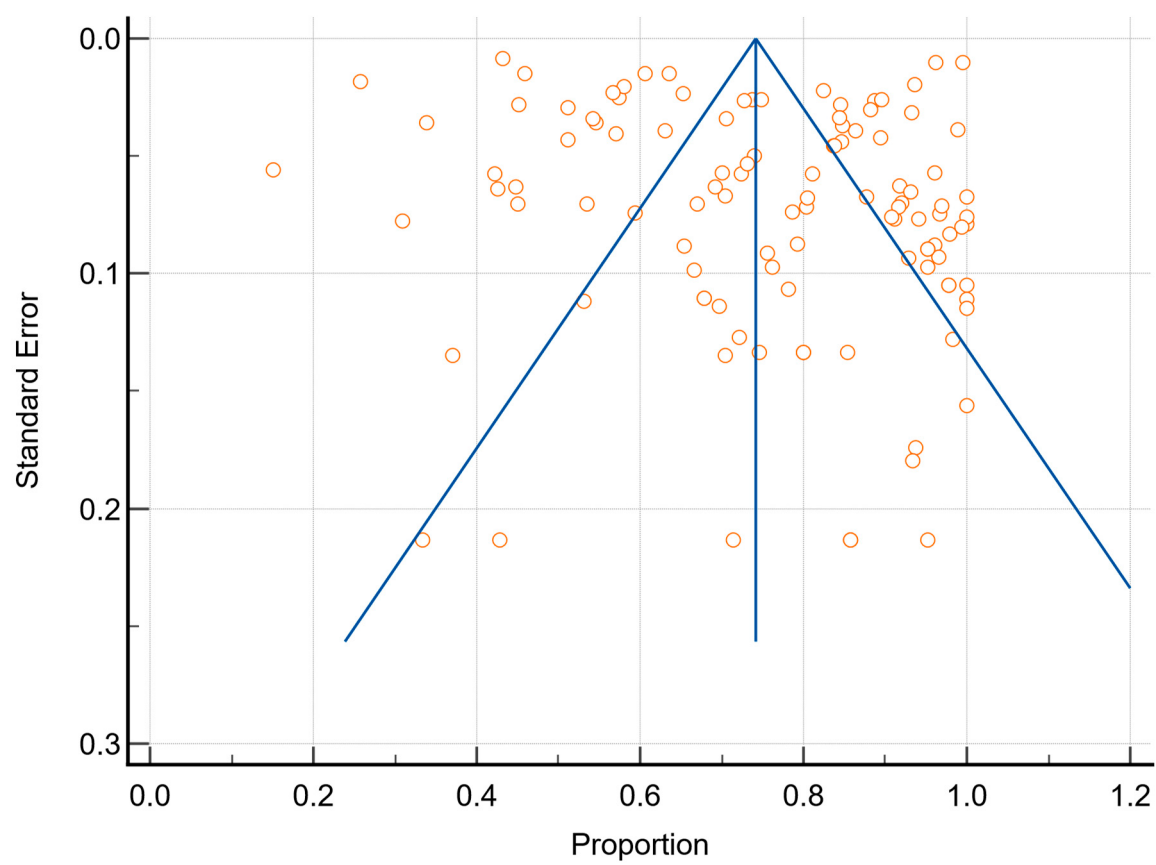

Supplement: Supplementary file 1 [file nutrients-18-01922-s001.zip › Supplemetary Figure S1 funnel plot v2.pdf]
